# A Conserved Upstream Motif Orchestrates Autonomous, Germline-Enriched Expression of *Caenorhabditis elegans* piRNAs

**DOI:** 10.1371/journal.pgen.1003392

**Published:** 2013-03-14

**Authors:** Allison C. Billi, Mallory A. Freeberg, Amanda M. Day, Sang Young Chun, Vishal Khivansara, John K. Kim

**Affiliations:** 1 Life Sciences Institute, University of Michigan, Ann Arbor, Michigan, United States of America; 2 Department of Human Genetics, University of Michigan, Ann Arbor, Michigan, United States of America; 3 Department of Computational Medicine and Bioinformatics, University of Michigan, Ann Arbor, Michigan, United States of America; 4 Department of Cellular and Molecular Biology, University of Michigan, Ann Arbor, Michigan, United States of America; The University of North Carolina at Chapel Hill, United States of America

## Abstract

Piwi-interacting RNAs (piRNAs) fulfill a critical, conserved role in defending the genome against foreign genetic elements. In many organisms, piRNAs appear to be derived from processing of a long, polycistronic RNA precursor. Here, we establish that each *Caenorhabditis elegans* piRNA represents a tiny, autonomous transcriptional unit. Remarkably, the minimal *C. elegans* piRNA cassette requires only a 21 nucleotide (nt) piRNA sequence and an ∼50 nt upstream motif with limited genomic context for expression. Combining computational analyses with a novel, in vivo transgenic system, we demonstrate that this upstream motif is necessary for independent expression of a germline-enriched, Piwi-dependent piRNA. We further show that a single nucleotide position within this motif directs differential germline enrichment. Accordingly, over 70% of *C. elegans* piRNAs are selectively expressed in male or female germline, and comparison of the genes they target suggests that these two populations have evolved independently. Together, our results indicate that *C. elegans* piRNA upstream motifs act as independent promoters to specify which sequences are expressed as piRNAs, how abundantly they are expressed, and in what germline. As the genome encodes well over 15,000 unique piRNA sequences, our study reveals that the number of transcriptional units encoding piRNAs rivals the number of mRNA coding genes in the *C. elegans* genome.

## Author Summary

Across the animal kingdom, Piwi-interacting small RNAs (piRNAs) protect genome integrity and promote fertility. While the functions of piRNAs are well-characterized, far less is known about how they are generated and how their expression is regulated. In the *Caenorhabditis elegans* genome, a conserved sequence motif lies upstream of many piRNA loci and appears to regulate their expression. We combined computational and experimental approaches to investigate the role of this motif in the expression of *C. elegans* piRNAs. We discovered that >70% of piRNAs are differentially enriched in male versus female germline, and these male and female piRNAs show different upstream motifs. Using a transgenic system for expressing synthetic piRNAs in vivo, we demonstrate that variation of a single nucleotide within this motif influences piRNA germline enrichment. We further show that the conserved motif is capable of driving piRNA expression in genomic isolation. Accordingly, the genomic distribution of these motifs determines which sequences are expressed as piRNAs in *C. elegans*. Our results suggest that each *C. elegans* piRNA represents an independent transcript whose sequence, abundance, and germline enrichment are encoded by a variant upstream motif, defining a novel modality for expression of piRNAs.

## Introduction

piRNAs and Piwi clade Argonautes arose in the primordial metazoan ancestor [1] and are generally restricted to the germline, where they act in an RNA-induced silencing complex (RISC) to silence foreign genetic elements. From protozoa to mammals, loss of Piwi proteins, and consequently piRNAs, results in abnormal fertility phenotypes or sterility, revealing their highly conserved and essential role in animal reproduction 2–8. piRNAs are incredibly diverse, with tens of thousands of unique sequences expressed in any single organism. While piRNAs in many organisms map to large, broadly syntenic genomic clusters, the sequences are not conserved among even closely related species, and no unifying sequence features have been identified beyond a bias among primary piRNAs for a 5′ uridine [9]–[15].

The mechanisms of de novo piRNA biogenesis remain elusive. In fly and mouse, primary piRNAs appear to be processed from long, single-stranded RNA precursors [9], [10], [14]. This long transcript is cleaved by the endoribonuclease Zucchini with little or no sequence specificity to generate candidate piRNA 5′ ends [16], [17], which are likely subsequently purified according to the binding preferences of the Piwi proteins that bind primary piRNAs [18]. Silkworm data suggest that the 3′ ends of these piRNA precursors are then trimmed by a 3′ to 5′ exonuclease until the 3′ end is sufficiently short for anchoring by Piwi to protect against further trimming [18]. The 3′ end is then methylated to prevent degradation [19]–[23]. While recent studies have shed light on the biogenesis of primary piRNAs in many animal models, little is known in any organism about how primary piRNA expression is regulated or how specific sequences are designated as piRNAs.

21U RNAs, a class of germline-enriched small RNAs, represent the piRNAs of *Caenorhabditis elegans*. They are terminally methylated [24]–[26], show a 5′ uridine bias [12], and are dependent upon and bound by the Piwi Argonaute PRG-1 [27], [28], which is required for normal fertility [3]. Yet *C. elegans* piRNAs exhibit some unusual features. While the vast majority of 21U RNAs map to two large genomic clusters on chromosome IV, the loci do not exhibit prominent strand biases [12]. The 21U RNAs also do not appear to play a prominent role in silencing transposable elements, a main function of mouse and fly piRNAs, nor do they engage a ping-pong amplification mechanism [27], [28]. Rather, PRG-1 and the 21U RNAs target aberrant and coding transcripts broadly via imperfect complementarity, triggering production of secondary endogenous siRNAs [27]–[31]. These 21U RNA-dependent 22G RNAs can induce chromatin changes to establish dominant, heritable target silencing [32]–[34]. 21U RNAs evolve rapidly, presumably constrained only by selection against sequences that silence mRNAs; thus, mismatch-tolerant 21U RNAs constitute an epigenetic memory of self versus non-self. Finally, a conserved motif lies upstream of 21U RNA genomic loci [12]. This stretch of sequence, which includes an eight-nucleotide (nt) core motif approximately 40 nt upstream of the 21U RNA locus, is conserved across divergent nematodes [12], [13]. Recently, Cecere et al. found that this motif is bound by forkhead family transcription factors and that deletion of the core motif abrogates 21U RNA expression [35], but it is still unknown how 21U RNA sequences are defined and how their expression is regulated.

Here, we demonstrate that piRNAs are expressed autonomously in *C. elegans*. Combining computational and transgenic approaches, we find that the conserved core motif defines the piRNA transcriptional cassette, specifying expression of 21U RNAs from genomic thymidines situated at an optimal distance downstream to determine which genomic sequences are expressed as *C. elegans* piRNAs. Core motifs also encode information dictating germline-specific expression of 21U RNAs. We show that more than 70% of *C. elegans* piRNAs are preferentially enriched in male or female germline. Unexpectedly, this germline enrichment appears to be enforced by a single nucleotide position within the core motif. We demonstrate autonomous expression of synthetic 21U RNAs from multiple minimal transgenic cassettes consisting only of the 8 nt core motif, the ∼40 nt intervening genomic spacer, the 21U RNA sequence, and ∼50–100 nt of flanking genomic context. Finally, we use single-copy transgenes integrated in genomic isolation to show that the clustered organization of endogenous piRNA loci is entirely dispensable for robust piRNA expression. Together, our results suggest that each 21U RNA locus encodes all of the information necessary for driving independent, autonomous transcription from more than 15,000 unique piRNA loci in *C. elegans*.

## Results

### A majority of 21U RNAs are male or female germline-enriched

**Figure 1:**
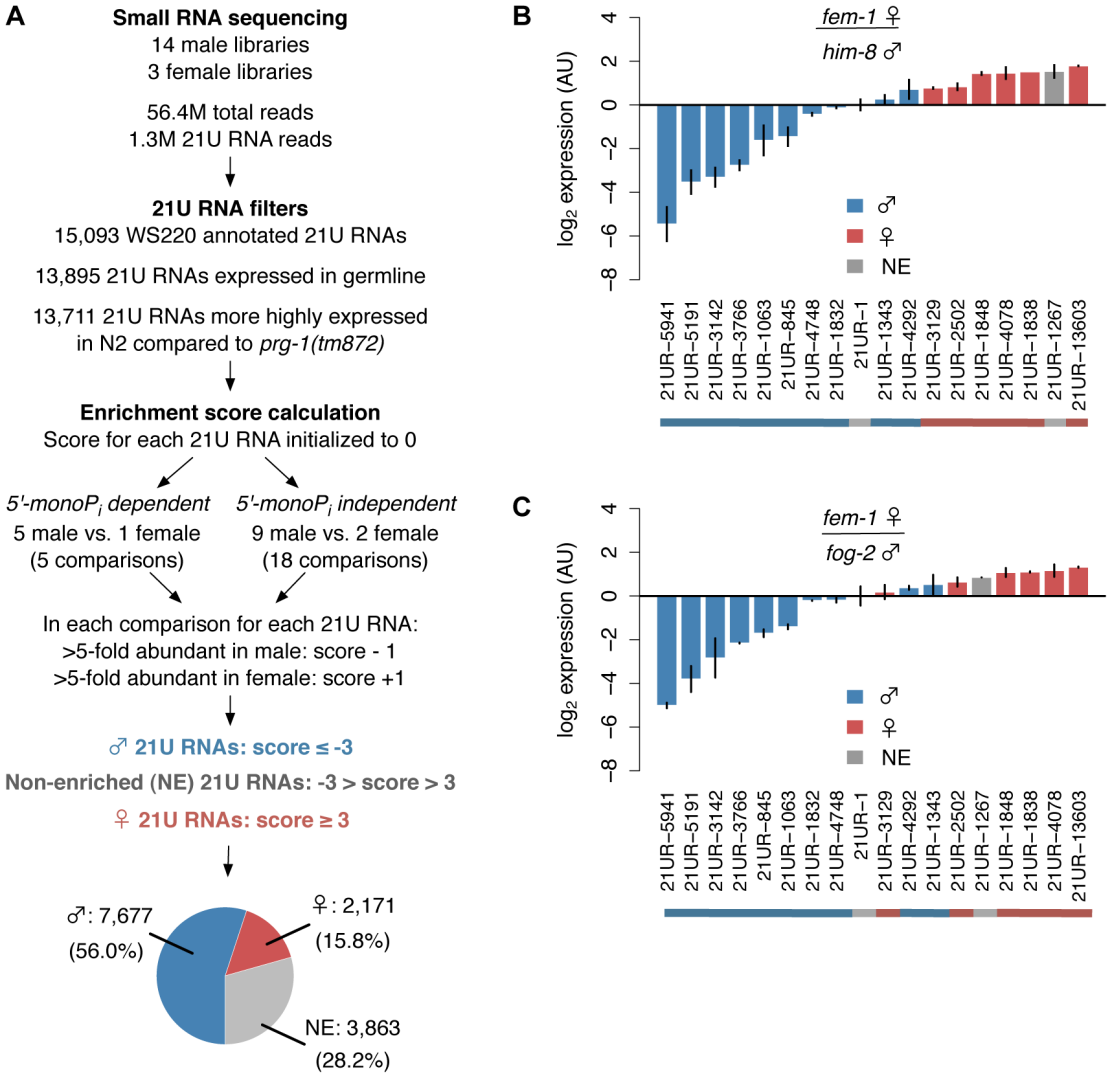
Over 70% of 21U RNAs show distinct germline enrichment. (A) Pipeline for computational identification of male and female 21U RNAs. A majority of 21U RNAs are classified as male or female germline-enriched. Pie chart depicts classification as proportion of 13,711 21U RNAs analyzed. (B,C) Male 21U RNAs are more highly expressed in male animals, and female 21U RNAs are more highly expressed in female animals. Relative expression of representative 21U RNAs was assayed by Taqman RT-qPCR in *him-8(e1489)* (B) and *fog-2(q71)* (C) male versus *fem-1(hc17)* female animals and normalized to non-enriched 21U RNA 21UR-1. Error bars: ±1 standard deviation (SD) of two biological replicates. AU: arbitrary units.

To investigate the mechanisms regulating piRNA expression, we first identified 21U RNA subclasses by performing a meta-analysis of over 50 million reads from published small RNA deep sequencing datasets [27], [36]–[42] (Table S1). Using the pipeline shown in Figure 1A, we determined that a majority of the 13,711 21U RNAs represented in our composite dataset show differential germline enrichment, distinguishing 7,677 (56.0%) unique male and 2,171 (15.8%) unique female germline-enriched 21U RNAs (hereafter, male and female 21U RNAs) (See Materials and Methods). The distribution of 21U RNA Enrichment scores is skewed toward the male (Figure S1A), whereas randomly generated 21U RNA count data show no significant skewing (Binomial test, p = 0.245) and define a false discovery rate below 1% (Figure S1B). To assess the reliability of the Enrichment score in classifying germline enrichment, we quantified the average relative abundance of every male 21U RNA between each pair of male and female libraries (Figure S1C); the reciprocal calculation was performed for female 21U RNAs (Figure S1D). On average, the abundance of male 21U RNAs is 6.8-fold higher in male libraries than female, whereas the abundance of female 21U RNAs is 2.4-fold higher in female libraries than male. Average abundance of 21U RNAs not classified as male or female (hereafter, non-enriched 21U RNAs) is approximately equal in male and female libraries (Figure S1E). Taqman RT-qPCR of select 21U RNAs in *fem-1(hc17)* adult female versus *him-8(e1489)* or *fog-2(q71)* adult male animals shows segregation of 21U RNAs according to germline enrichment classification (Figure 1B, 1C), endorsing our computational discovery of germline-enriched piRNA subclasses in *C. elegans*.

### Male and female 21U RNAs show different expression profiles in embryo

Our meta-analysis also revealed a subpopulation of 21U RNAs highly abundant in embryo. Comparison of the abundances of male and female 21U RNAs in mixed stage embryo sequencing libraries showed that female 21U RNAs were overrepresented in embryo relative to male. A higher proportion of unique female 21U RNAs were detected in embryo (χ^2^ test, p = 9.2e−45) (Figure S2A, S2B). Furthermore, unique female 21U RNAs were on average 4.4-fold more abundant in embryo than unique male species (Welch's *t*-test, p = 3.4e−148). The trend is corroborated by Taqman analysis showing depletion of male 21U RNAs and enrichment of female 21U RNAs in embryo (Figure S2C–S2E). These data suggest that female piRNAs are preferentially inherited into *C. elegans* embryo, consistent with previous observations in fly [43]–[45]. Parallel classification and embryonic enrichment analysis of 26G RNAs, germline-enriched primary endo-siRNAs, recapitulated previously observed inheritance patterns [38] and validated the ability of our pipeline to identify germline-enriched small RNA subclasses (Figure S2F, S2G).

### Male 21U RNA targets reflect spermatogenic gonad restriction

21U RNAs target transcripts with imperfect complementarity of up to three mismatches to trigger production of antisense 22G RNAs proximal to the targeting site [29], [30], [32], [33]. The lax complementarity requirement for piRNA-mediated silencing predicts widespread targeting capacity. Compartmentalization of piRNA expression to the male and female germline may help to confer specificity. To investigate the biological significance of germline-enriched 21U RNA subclasses, we first examined whether male and female 21U RNAs target distinct subsets of genes. We analyzed the overlap between their respective dependent 22G RNAs by identifying 22G RNAs that map antisense to within 40 nt of 21U RNA target sites [30] (See Materials and Methods). Ignoring 22G RNAs detected in *prg-1(n4357)* deep sequencing datasets, as these are likely not 21U RNA-dependent, we identified 11,377 (72.3%) unique 22G RNAs that are likely male 21U RNA-dependent and 3,855 (24.5%) unique 22G RNAs that are likely female 21U RNA-dependent (Figure S3A). Only 494 (3.1%) unique 22G RNAs lie within 40 nt of both a male and female 21U RNA target site, precluding assignment to either category. This overlap is less than expected when 22G RNAs from random but similarly sized sets of 21U RNAs are compared (χ^2^ test, p = 0.012). We then compared the 5,956 male and 1,387 female 21U RNA targets identified in young adult [29] and gravid [30] animals, respectively. Overlap between targets (149 overlapping targets) is significantly lower compared to random sets of genes (294 overlapping and 6,756 non-overlapping targets; χ^2^ test, p = 7.7e−13) (Figure S3B).

Because targets of 21U RNAs are subject to transgenerational silencing [32]–[34], 21U RNAs are unlikely to evolve to target transcripts required in the germline. Similarly, male 21U RNAs would not be expected to target transcripts required for spermatogenesis; however, temporal separation of the spermatogenic and oogenic gonads might permit evolution of male 21U RNAs capable of targeting transcripts required for oogenesis. We examined our data for evidence of this evolutionary signature. As comprehensive lists of genes required for spermatogenesis and oogenesis have yet to be assembled, we used as a proxy lists of transcripts identified by microarray studies as enriched during spermatogenesis (865 transcripts) or oogenesis (1,030) [46]. Comparing male 21U RNA targets to randomly generated gene lists, we found that male 21U RNA targets are indeed depleted of spermatogenesis transcripts (χ^2^ test, p = 0.044), but neither enriched nor depleted for oogenesis transcripts (χ^2^ test, p = 0.76) (Figure S3C). Curiously, we do not observe the same signature for female 21U RNAs (Figure S3D). Their targets are neither enriched nor depleted for spermatogenesis transcripts (χ^2^ test, p = 0.27), as expected, but female 21U RNA targets are significantly enriched for oogenesis transcripts (χ^2^ test, p = 0.0017). These differences between male and female 21U RNA targeting suggest that the evolutionary pressures acting on male and female 21U RNA sequences may differ (See Discussion).

### Male and female 21U RNAs have distinct core upstream motifs

**Figure 2:**
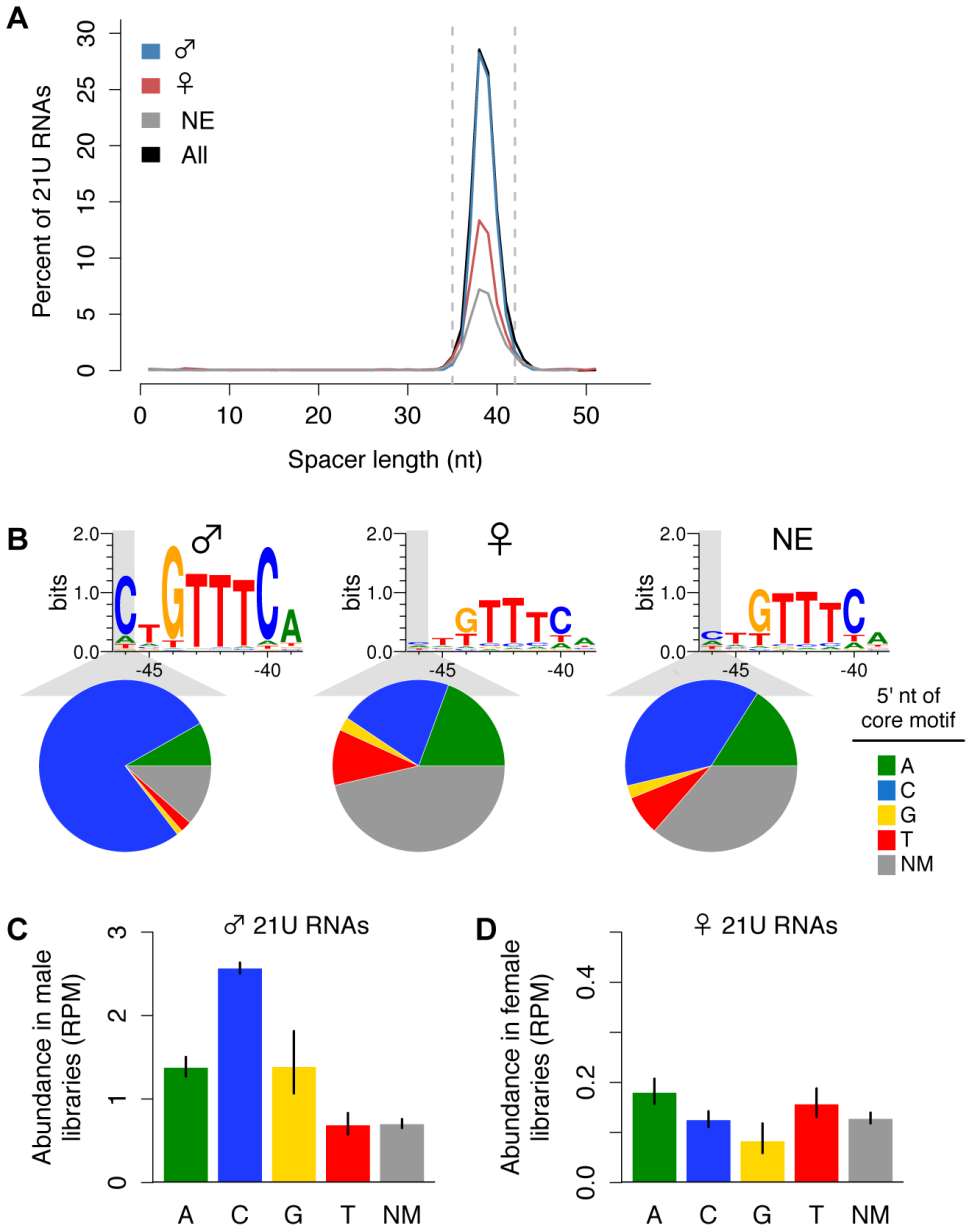
Variation in the core upstream motif correlates with 21U RNA germline enrichment. (A) Spacer lengths follow expected distribution for all enrichment classifications. Dotted lines: canonical spacer length range (35–42 nt). (B) Male, but not female, 21U RNA loci show enrichment for core motifs with 5′ cytidines. Significantly fewer female 21U RNAs exhibit a GTTTC-containing core motif than male. Top: Weblogo plots illustrate core motif differences. Bottom: Pie charts depict proportions of 21U RNAs with GTTTC-containing core motifs indicating the 5′ nt (colors) or with no GTTTC-containing core motif (NM, no motif, dark grey). (C) Core motif variations correlate with male 21U RNA abundance in 5′-monophosphate-dependent libraries. Average 21U RNA abundance was calculated based on the 5′ nt of the core motif. Error bars: ±1 standard error of the mean (SEM). (D) Core motif variations do not correlate with female 21U RNA abundance in 5′-monophosphate-dependent libraries. Average 21U RNA abundance was calculated as in (C).

To investigate how 21U RNA germline enrichment information is genetically encoded, we analyzed the genomic loci of the 13,387 21U RNAs that map uniquely to the genome. Comparison of male and female 21U RNA sequences identified no differences in content; therefore, we evaluated the 21U RNA upstream region. The 8 nt core motif, with consensus sequence CTGTTTCA, is separated from the 21U RNA locus by an A/T-rich spacer of ∼35 to 42 nt [12]. Scanning the 60 nt upstream of each 21U RNA for the best conserved central GTTTC of the core motif, we found that 6,615 of 7,677 (88%) male 21U RNAs show a canonical, GTTTC-containing core motif, compared to only 1,119 of 2,171 (54%) female 21U RNAs. While the length of the A/T-rich spacer does not differ between male and female 21U RNAs (Figure 2A), core motif sequence analysis revealed a striking difference: only the core motifs of male 21U RNAs are enriched for a 5′ cytidine. 5,765 of 7,677 (77%) male 21U RNAs are located downstream of canonical, GTTTC-containing core motifs with a 5′ cytidine, compared to only 443 of 2,171 (21%) female 21U RNAs (χ^2^ test, p = 7.9e-137) (Figure 2B). To examine whether this 5′ core motif position influences 21U RNA expression, we calculated the average abundance of male and female 21U RNAs grouped by 5′ core motif nt. Male 21U RNAs with 5′ cytidine core motifs are significantly more abundant than all other male 21U RNAs (Figure 2C, Welch's *t*-test p-values in Table S2), consistent with the previous observation that 21U RNAs whose core motifs better match the consensus sequence are more highly expressed [12]. No other subgroup differs significantly in abundance from all others among the male, female, and non-enriched 21U RNAs (Figure 2C and 2D, Table S2), suggesting that GTTTC-containing core motifs with a 5′ cytidine are overrepresented among male 21U RNAs and may drive male germline expression.

### A transgenic synthetic 21U RNA recapitulates features of endogenous 21U RNAs

**Figure 3:**
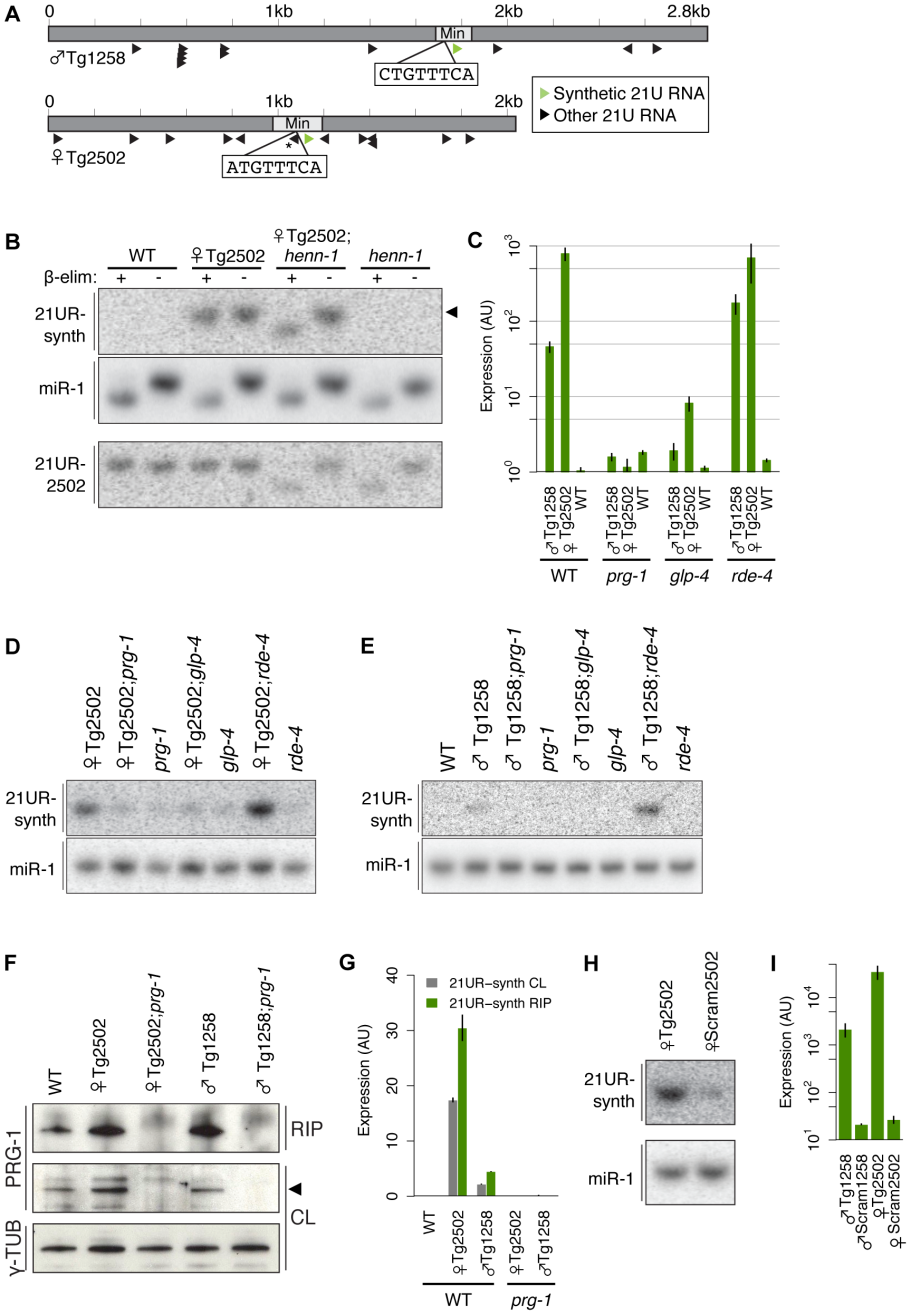
A transgenic synthetic 21U RNA shows characteristics of endogenous 21U RNAs. (A) Diagram of Tg (dark grey) and Min (light grey) transgenes with core motif sequences shown. Asterisk indicates a 21U RNA whose core motif is disrupted by 21UR-synth and is therefore predicted not to express. (B) 21UR-synth is methylated by HENN-1. 21UR-synth is specifically detected in transgenic strains and is susceptible to β-elimination only in the *henn-1(tm4477)* background. Arrowhead represents migration of a 21 nt size marker. 21UR-synth blot was reprobed for miR-1. Endogenous ♀21UR-2502 is shown as a control. (C–E) 21UR-synth is a *prg-1*-dependent, germline-enriched 21U RNA. 21UR-synth detection by Taqman RT-qPCR (C) and northern blot (D,E) is greatly decreased in *prg-1(tm872)* and *glp-4(bn2)* germline-deficient mutant animals, but intact in *rde-4(ne301)* mutant animals. Error bars: ±1 SD of three biological replicates. (F) anti-PRG-1 antibody immunopurifies PRG-1 complexes. CL: crude lysate, RIP: RNA immunoprecipitation. (G) 21UR-synth is bound by endogenous PRG-1. Error bars: ±1 SD of two technical replicates; data are representative of two independent experiments. (H,I) Loss of the core motif dramatically decreases 21UR-synth expression by northern blot (H) and Taqman qRT-PCR (I). Error bars: ±1 SD of three biological replicates.

To explore the significance of variation at the 21U RNA upstream motif, we developed a transgenic system to express synthetic 21U RNAs from high-copy, integrated arrays in vivo (Figure 3A). 2–3 kilobase regions of genomic sequence from a chromosome IV piRNA cluster were cloned, and a central 21U RNA (male 21U RNA ♂21UR-1258 or female 21U RNA ♀21UR-2502) was mutated to a unique synthetic 21 nt sequence (21UR-synth) to distinguish transgenic from endogenous expression. The sequences were then further mutated to generate the panel of transgenes shown in Table 1. Transgenes are named for the endogenous 21U RNA replaced by 21UR-synth, with prefixes to indicate transgene type (e.g., ♀Tg2502 represents the otherwise wild-type transgene encoding 21UR-synth in place of ♀21UR-2502). These transgenes are carried by the vector pCFJ178 [47], which also expresses the *C. briggsae unc-119* gene (Figure S4A), enabling gross normalization for variable array expression.

**Table 1:** Descriptions of transgenic alleles and features of the transgenes.

High-copy transgenes								
Transgene name	Transgene description	Size (kb)	Upstream motif	1^st^ nt	Chr	Allele	Strain	Backgrounds
♂Tg1258	Cluster with synthetic 21U RNA replacing 21UR-1258	2.87	attaagcCTGTTTCAcattttt	U	V	*xkIs1*	QK7	WT, *prg-1*, *rde-4*, *glp-4*, *him-8*, *fem-1*, *henn-1*
♂Scram1258	♂Tg1258 with upstream motif scrambled	2.87	atta**ttcagaccgtcta**ttttt	U	N/D	*xkIs11*	QK8	WT
♂C>A1258	♂Tg1258 with upstream motif first nt mutated C>A	2.87	attaagc**A** TGTTTCAcattttt	U	II	*xkIs6*	QK9	WT, *prg-1*, *him-8*, *fem-1*
♂21U>A1258	♂Tg1258 with synthetic 21U RNA first nt mutated U>A	2.87	attaagcCTGTTTCAcattttt	**A**	N/D	*xkIs14*	QK10	WT
♂21U>G1258	♂Tg1258 with synthetic 21U RNA first nt mutated U>G	2.87	attaagcCTGTTTCAcattttt	**G**	N/D	*xkIs15*	QK11	WT
♂Min1258	Minimal 21U RNA construct from ♂Tg1258	0.17	attaagcCTGTTTCAcattttt	U	N/D	*xkIs16*	QK12	WT, *prg-1*, *rde-4*, *glp-4*
♀Tg2502	Cluster with synthetic 21U RNA replacing 21UR-2502	2.04	aaataaaATGTTTCAactagtc	U	I	*xkIs5*	QK13	WT, *prg-1*, *rde-4*, *glp-4*, *him-8*, *fem-1*, *henn-1*
♀Scram2502	♀Tg2502 with upstream motif scrambled	2.04	aaataaa**ggacacttattat**tc	U	N/D	*xkIs12*	QK14	WT
♀A>C2502	♀Tg2502 with upstream motif first nt mutated C>A	2.04	aaataaa**C** TGTTTCAactagtc	U	X	*xkIs10*	QK15	WT, *prg-1*, *him-8*, *fem-1*
♀21U>A2502	♀Tg2502 with synthetic 21U RNA first nt mutated U>A	2.04	aaataaaATGTTTCAactagtc	**A**	N/D	*xkIs17*	QK16	WT
♀21U>G2502	♀Tg2502 with synthetic 21U RNA first nt mutated U>G	2.04	aaataaaATGTTTCAactagtc	**G**	N/D	*xkIs18*	QK17	WT
♀Min2502	Minimal 21U RNA construct from ♀Tg2502	0.23	aaataaaATGTTTCAactagtc	U	N/D	*xkIs19*	QK18	WT, *prg-1*, *rde-4*, *glp-4*
♂Min1415	Minimal 21U RNA construct generated from 21UR-1415	0.22	ttttcgcCTGTTTCAaggagtt	U	N/D	*xkIs20*	QK19	WT
♂MinC>A1415	♂Min1415 with upstream motif first nt mutated C>A	0.22	ttttcgc**A** TGTTTCAaggagtt	U	N/D	*xkIs21*	QK20	WT
Tg1415–Tg2109	Minimal 21U RNA construct with 21UR-synth replacing 21UR-1415 and 21UR-synthB replacing 21UR-2109	0.26	ttttcgcCTGTTTCAaggagtt taatctcCTGTTTCAcaatatt	U	N/D	*xkIs22*	QK21	WT
Scram1415-Tg2109	Tg1415–Tg2109 with 21UR-1415 upstream motif scrambled	0.26	ttttcgtagg**taccc** **tgtag**tt taatctcCTGTTTCAcaatatt	U	N/D	*xkIs23*	QK22	WT

MosSCI transgenes								
Transgene name	Transgene description	Size (kb)	Upstream motif	1^st^ nt	Chr	Allele	Strain	
♂Tg1258	Cluster with synthetic 21U RNA replacing 21UR-1258	2.87	attaagcCTGTTTCAcattttt	U	IV	*xkSi3*	QK23	
♂Scram1258	♂Tg1258 with upstream motif scrambled	2.87	atta**ttcagaccgtcta**ttttt	U	IV	*xkSi23*	QK24	
♂C>A1258	♂Tg1258 with upstream motif first nt mutated C>A	2.87	attaagc**A** TGTTTCAcattttt	U	IV	*xkSi17*	QK25	
♀Tg2502	Cluster with synthetic 21U RNA replacing 21UR-2502	2.04	aaataaaATGTTTCAactagtc	U	IV	*xkSi13*	QK26	
♀Scram2502	♀Tg2502 with upstream motif scrambled	2.04	aaataaa**ggacacttattattc**	U	IV	*xkSi28*	QK27	
♀A>C2502	♀Tg2502 with upstream motif first nt mutated C>A	2.04	aaataaa**C** TGTTTCAactagtc	U	IV	*xkSi20*	QK28	
♀Min2502	Minimal 21U RNA construct from ♀Tg2502	0.23	aaataaaATGTTTCAactagtc	U	II	*xkSi30*	QK29	

Both high-copy and MosSCI transgenes used in this study are listed with a short description, sequence characteristics, integration information, and strain notation. Full transgene data are listed in Materials and Methods. Bolded letters indicate mutated nucleotides. Eight nt core upstream motifs are capitalized while motif positions are underlined. N/D, not determined.

IV. The ♀Min2502 transgene also expresses from a single-copy insertion on chromosome II.

To validate our transgenic system, we examined whether 21UR-synth recapitulates all of the known features and genetic sensitivities of endogenous 21U RNAs. 21U RNAs are 2′-O-methylated at the 3′ terminus by the conserved methyltransferase HENN-1 [24]–[26]. Northern blot for 21UR-synth in transgenic strains identified a 21 nt species that is terminally methylated in a *henn-1*-dependent manner (Figure 3B). Robust, specific detection of the 3′ terminus by Taqman RT-qPCR [48] confirms that this species corresponds to 21UR-synth (Figure 3C). Levels of endogenous 21U RNAs ♂21UR-1258 and ♀21UR-2502 are largely unaffected by expression of the transgenes (Figure S4B). Endogenous 21U RNAs are generated in the germline and require PRG-1 for accumulation [27], [28]. Accordingly, 21UR-synth is highly depleted by loss of *prg-1* and in the *glp-4(bn2)* germline-deficient mutant (Figure 3C–3E). 21UR-synth and endogenous 21U RNAs are also specifically detected in immunoprecipitated PRG-1 complexes, while a microRNA control is not (Figure 3F and 3G, Figure S5). To rule out the unlikely possibility that transgenic products corresponding to the 21UR-synth sequence might be generated by an alternative, Dicer-dependent mechanism, we assayed 21UR-synth accumulation in a null mutant of *rde-4*. This gene encodes a dsRNA binding protein that is a key cofactor of Dicer in siRNA biogenesis [49]–[51], but dispensable for 21U RNA production (Figure S4B). Loss of *rde-4* does not impair 21UR-synth expression (Figure 3C–3E), suggesting that 21UR-synth does not represent an siRNA generated from the high-copy transgenic array.

Finally, we examined whether the core motif is required for 21UR-synth expression. We scrambled the core motif to eliminate any resemblance to the consensus sequence (♂Scram1258 and ♀Scram2502 transgenes; Table 1). 21UR-synth levels in these strains are depleted by more than 100-fold after normalization for array expression (Figure 3H, 3I), consistent with previous findings that deletion of the core motif depletes 21U RNA expression [35]. Together, these data demonstrate that 21UR-synth represents a bona fide 21U RNA and support the use of this transgenic system for exploring 21U RNA biology in vivo.

### 21U RNA core upstream motif variation influences germline enrichment

**Figure 4:**
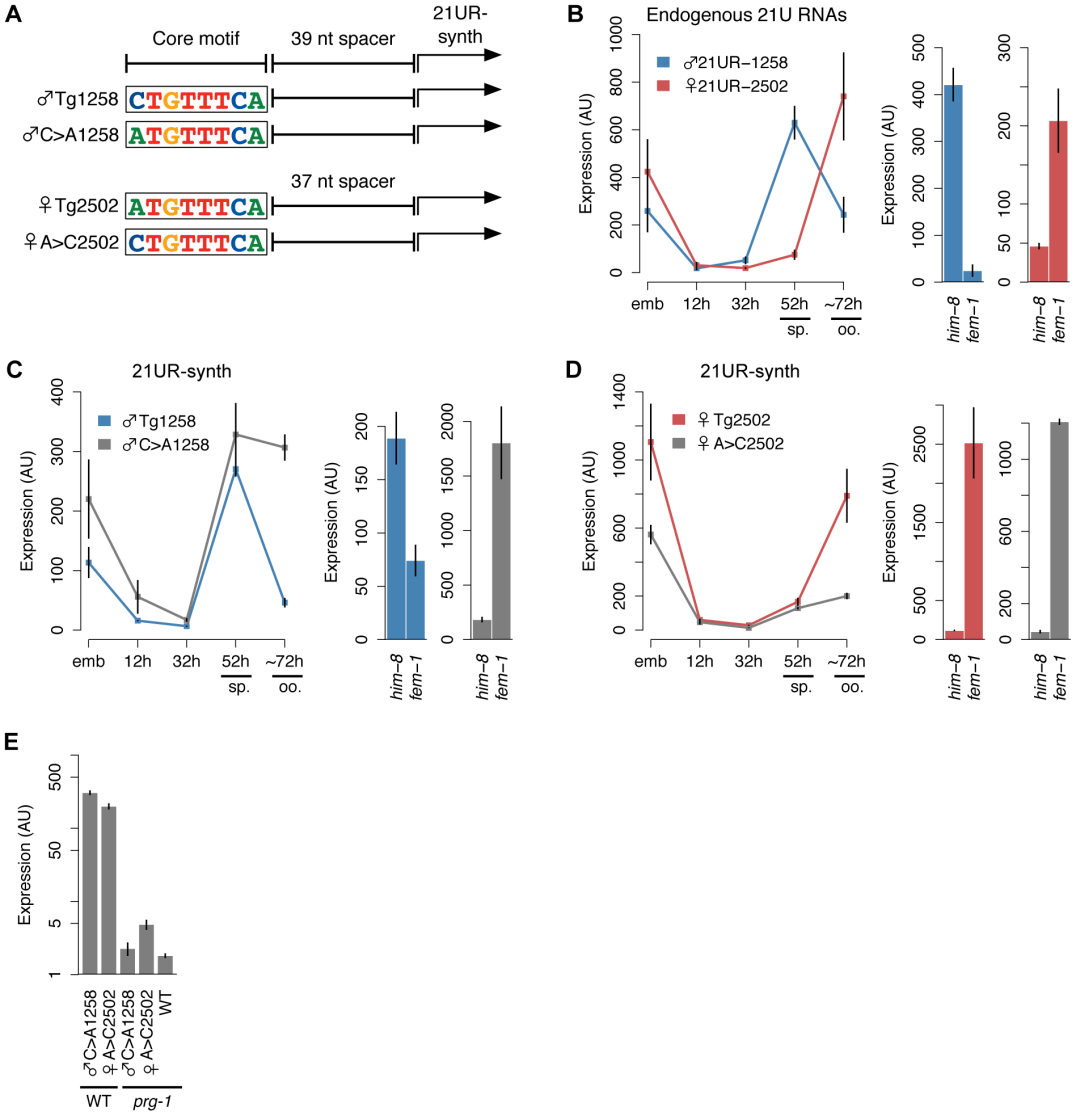
A 5′ cytidine in the core upstream motif promotes male germline expression pattern of 21UR-synth. (A) Schematic of transgenes with 5′ nt of core motif mutated. (B) Left: Endogenous ♂21UR-1258 and ♀21UR-2502 peak during spermatogenesis (sp.) and oogenesis (oo.), respectively. Right: Germline enrichment patterns are recapitulated in *him-8(e1489)* male and *fem-1(hc17)* female animals. Error bars: ±1 SD of three biological replicates. (C) The male expression pattern of 21UR-synth from ♂Tg1258 is disrupted by core motif mutation in ♂C>A1258. Error bars: ±1 SD of three biological replicates. (D) The female expression pattern of 21UR-synth from ♀Tg2502 is disrupted by core motif mutation in ♀A>C2502, but expression in *fem-1(hc17)* female is not lost. Error bars: ±1 SD of three biological replicates. (E) Mutating the 5′ nt of the core motif does not affect 21UR-synth *prg-1* dependence.

We then used our transgenic system to test whether variation at the core motif 5′ position affects germline expression of 21UR-synth (Figure 4A). Endogenous male 21U RNA ♂21UR-1258, which lies downstream of a **C**TGTTTCA core motif, peaks in expression during spermatogenesis (52 h time point) and is highly expressed in *him-8(e1489)* male adult; in contrast, expression of endogenous ♀21UR-2502, with an **A**TGTTTCA core motif, peaks after the spermatogenesis-to-oogenesis transition in adulthood (∼72 h) and is highly expressed in *fem-1(hc17)* female adult (Figure 4B). Accordingly, the ♂Tg1258 and ♀Tg2502 transgenes express 21UR-synth in similar male and female patterns, respectively (Figure 4C and 4D, colored lines/bars). Toggling the core motif from **C**TGTTTCA to **A**TGTTTCA (♂C>A1258 transgene) or **A**TGTTTCA to **C**TGTTTCA (♀A>C2502) disrupts these germline-specific expression patterns. Whereas 21UR-synth expression from ♂Tg1258 plummets after spermatogenesis, loss of the core motif 5′ cytidine in the ♂C>A1258 transgenic strain results in sustained 21UR-synth expression through oogenesis; the ♂C>A1258 transgene also preferentially expresses 21UR-synth in *fem-1(hc17)* female (Figure 4C). Thus, mutating the 5′ cytidine of a male 21U RNA core motif results in a failure to restrict 21U RNA expression to spermatogenesis. Similarly, introducing a 5′ cytidine into a female 21U RNA core motif impairs restriction of expression to oogenesis: while ♀Tg2502 expression of 21UR-synth increases dramatically during the spermatogenesis-to-oogenesis transition, gain of the motif 5′ cytidine in the ♀A>C2502 transgene dampens this increase (Figure 4D). These results suggest that this single nucleotide orchestrates the accurate switching of 21U RNA expression in the hermaphroditic germline. However, 21UR-synth expression from the ♀A>C2502 transgene is still high in *fem-1(hc17)* female, indicating that other elements contribute to female 21U RNA expression patterns. This is consistent with our finding that female 21U RNA core motifs show no bias at the 5′ nucleotide, and indeed ∼21% of female 21U RNA core motifs show a 5′ cytidine (Figure 2B). As expected, 21UR-synth expression from the ♂C>A1258 and ♀A>C2502 transgenes is still dependent upon *prg-1* (Figure 4E).

### A 5′ thymidine is required for robust expression from the 21UR-synth locus

**Figure 5:**
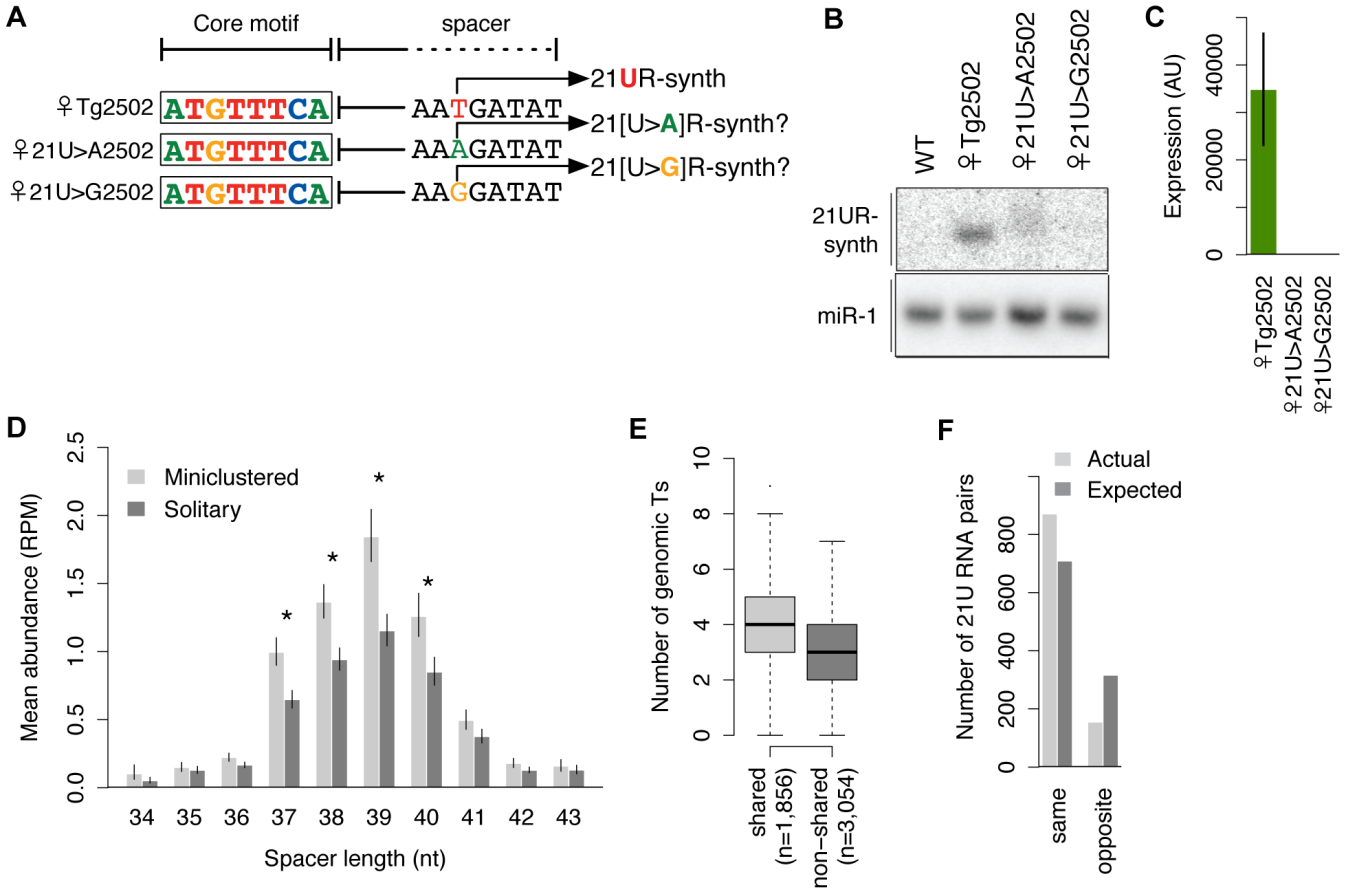
21U RNA sequences are specified by the genomic positions of upstream core motifs. (A) Schematic of transgenes with 5′ nt of 21U RNA mutated. (B–C) Mutation of the 5′ genomic thymidine disrupts expression of 21UR-synth by northern blot (B) and Taqman assay (C). (D) 21U RNA abundances correlate with distances downstream of core motifs. Miniclustered 21U RNAs with 37–40 nt spacer lengths are more abundant than solitary 21U RNAs. Asterisks indicate Welch's *t*-tests, p<0.05. Error bars: ±1 SEM. (E) Optimal downstream windows are more thymidine-rich for shared core motifs than non-shared (Welch's *t*-test, p = 2.5e-46). The number of genomic thymidines located 35–42 nt downstream of each GTTTC-containing motif was counted. (F) 21U RNA miniclusters are significantly biased for being composed of 21U RNAs with the same, as opposed to opposite, germline enrichment than expected if the same 21U RNAs were randomly paired.

It is not yet known how individual genomic sequences are selected for expression as piRNAs. As the core motifs, but not the sequences, of 21U RNAs are conserved across *Caenorhabditis* species, it seemed possible that the core motifs themselves might determine what sequences are expressed as 21U RNAs by directing their expression from genomic thymidines located an optimal distance downstream. We explored this hypothesis by mutating the genomic thymidines encoding the first nucleotide of 21UR-synth to adenosine (21U>A transgenes) or guanosine (21U>G transgenes), such that the transgenes encode 21[U>A]R-synth or 21[U>G]R-synth, respectively (Figure 5A, Figure S6A). These putative products emulate the 5′ nucleotide identity of microRNAs (predominantly 5′ uridine and adenosine) and endo-siRNAs (predominantly 5′ guanosine). Small RNAs expressed from these transgenes and recognized by the 21UR-synth northern blot probe differ in size from and are less abundant than wild-type 21UR-synth (Figure 5B, Figure S6B). By Taqman analysis, 21[U>A]R-synth and 21[U>G]R-synth are detected at levels more than 150-fold lower than 21UR-synth after normalization for array expression (Figure 5C, Figure S6C), suggesting that 21[U>A]R-synth and 21[U>G]R-synth are poorly transcribed, stabilized, or both.

### The genomic positioning of core motifs specifies 21U RNA sequences

We hypothesized that 21U RNA expression from a particular genomic thymidine may simply be a function of distance from a core motif (i.e., length of the intervening genomic spacer). Therefore, the presence of multiple thymidines within the optimal genomic window downstream of a core motif might result in expression of multiple, overlapping 21U RNAs. Indeed, many *C. elegans* piRNAs map to proximal genomic thymidines as members of “miniclusters” of overlapping 21U RNAs that appear to share an upstream core motif. To explore the relationship between core motif position and expression, we extracted read count information from deep sequencing of wild-type adult animals [27] for uniquely mapping 21U RNAs and analyzed their corresponding genomic loci. After separating 21U RNAs into those that share a core motif with at least one other uniquely mapping 21U RNA (“miniclustered”; 4,550 21U RNAs) and those that do not (“solitary”; 8,837 21U RNAs), we grouped 21U RNAs by length of genomic spacer and examined their abundance. For both miniclustered and solitary 21U RNAs, the resulting distributions peak at a 39 nt spacer length and decrease as the spacer lengthens or shortens (Figure 5D). The evident correlation between spacer length and robustness of expression explains previous observations that miniclustered 21U RNAs routinely show great variation in abundance [39].

We also observed that miniclustered 21U RNAs with 37–40 nt spacers are more abundant than solitary 21U RNAs at matched positions (Figure 5D, asterisks), suggesting that 21U RNA miniclusters may arise when expression is driven more robustly. To investigate this further, we compared the core motifs associated with miniclustered 21U RNAs (“shared” motifs) versus solitary 21U RNAs (“non-shared” motifs). We found that a significantly larger proportion of miniclustered 21U RNAs (3,580 of 4,550, 79%) than solitary 21U RNAs (5,667 of 8,837, 64%) are associated with canonical, GTTTC-containing core motifs (χ^2^ test, p = 1.4e−66). Additionally, we observed significantly greater thymidine richness in the optimal genomic windows 35–42 nt downstream of shared GTTTC-containing motifs versus non-shared (Welch's *t*-test, p = 4.0e−95) (Figure 5E). Therefore, particular sequences of 21U RNAs may not be specified intrinsically; rather, core motifs may simply direct expression of 21U RNAs from one or more downstream thymidines, depending on the strength of the motif and the number of optimally positioned thymidines.

To further confirm the association between the core motif and germline enrichment, we analyzed miniclusters consisting of two germline-enriched 21U RNAs (1,026 pairs). Random assortment of these 21U RNAs would predict 66% male∶male, 4% female∶female, and 31% male∶female pairs; however, we observed 73% male∶male, 12% female∶female, and only 15% male∶female pairs. Thus, 85% of pairs showed matching enrichment classification (Figure 5F), a significant departure from the 69% expected by random assortment (χ^2^ test, p = 9.6e−28). We note that this paucity of mixed male∶female 21U RNA miniclusters likely contributes to the low number of 22G RNAs that can be attributed to both male and female 21U RNAs (Figure S3A).

### Each upstream motif and 21U RNA sequence constitutes a tiny, autonomous transcriptional unit

**Figure 6:**
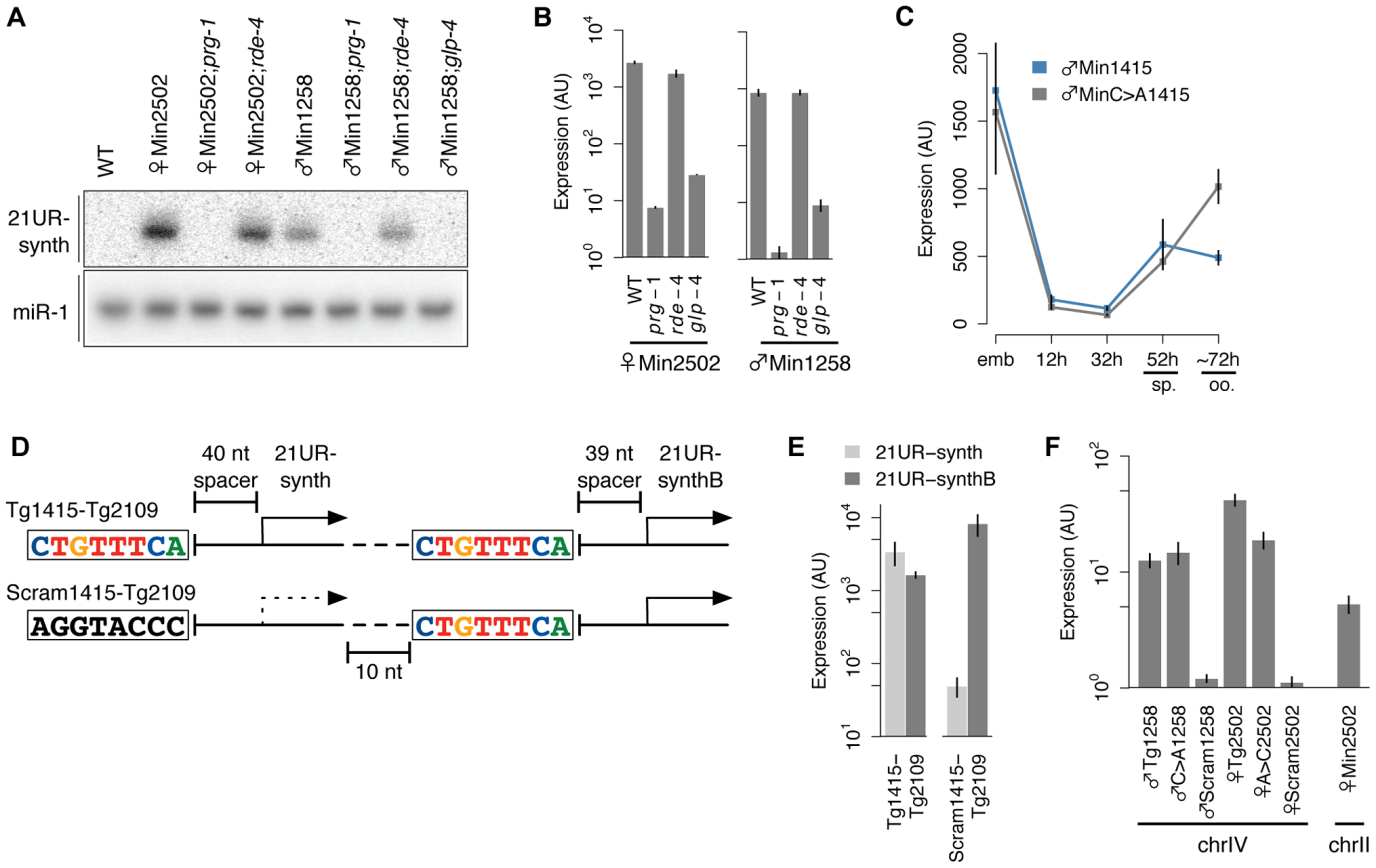
21U RNAs represent independent transcriptional units. (A–B) 21UR-synth expressed from a minimal transcriptional cassette shows *prg-1* dependence, *rde-4* independence, and germline enrichment by northern blot (A) and Taqman assay (B). (C) The male expression pattern of 21UR-synth from ♂Min1415 is disrupted by core motif mutation in ♂MinC>A1415. Error bars: ±1 SD of three biological replicates. (D) Schematic of transgenes encoding two closely adjacent 21U RNAs. (E) Scrambling the core motif upstream of 21UR-synth abrogates 21UR-synth, but not 21UR-synthB, expression levels. (F) The ♂Tg1258, ♂C>A1258, ♀Tg2502, and ♀A>C2502 transgenes, but not the ♂Scram1258 or ♀Scram2502 transgenes, express from single copy insertions on chromosome.

The absence of long, unidirectional 21U RNA clusters in the *C. elegans* genome and the presence of the conserved upstream motif have generated speculation that 21U RNAs represent autonomously transcribed units [12], [27], [28]. This is further suggested by our and others' findings that scrambling or deleting the core motif abrogates 21U RNA expression (Figure 3H, 3I and [35]). To test whether 21U RNAs express independently, we generated transgenes representing putative minimal 21U RNA transcriptional units. Each of these Min transgenes encodes only a single core motif, spacer, and 21U RNA, with limited 5′ and 3′ genomic context (Figure 3A). Strikingly, 21UR-synth expressed from this minimal context shows the same size, *prg-1* dependence, *rde-4* independence, and germline enrichment as endogenous 21U RNAs (Figure 6A, 6B), indicating that the sequence features conferring these 21U RNA characteristics are contained within a single 21U RNA transcriptional unit. To ensure that the 5′ nucleotide of the core motif still influences germline enrichment within this minimal context, we also generated and tested an independent set of minimal 21UR-synth transgenes with core motif intact (♂Min1415) or first nucleotide toggled (♂MinC>A1415). These transgenes also showed impaired male germline enrichment upon toggling of the core motif 5′ nucleotide (Figure 6C), reaffirming our conclusions that a core motif 5′ cytidine helps to orchestrate 21U RNA male germline enrichment.

### The 21U RNA transcriptional unit is autonomous

To explore the autonomy of the 21U RNA transcriptional unit further, we generated additional transgenes carrying <300 nt of genomic sequence encoding two adjacent 21U RNA transcriptional units on the same strand (Figure 6D). To create the “wild-type” Tg1415-Tg2109 transgene, the upstream 21U RNA locus, corresponding to 21UR-1415, was mutated to encode 21UR-synth, and the downstream locus, corresponding to 21UR-2109, was mutated to encode a different unique synthetic 21U RNA (21UR-synthB). We then scrambled the core motif of the upstream 21U RNA locus to generate the Scram1415-Tg2109 transgene and measured relative expression of the two synthetic 21U RNAs from each transgene. Much as expression of 21UR-synth is vastly decreased by loss of the core motif in the ♂Scram1258 and ♀Scram2502 transgenes above (Figure 3H, 3I), 21UR-synth is expressed at far lower levels than 21UR-synthB from the Scram1415-Tg2109 transgene, whereas expression of the synthetic 21U RNAs from the Tg1415-Tg2109 transgene is comparable (Figure 6E). This experiment specifically pursues a recent finding by Cecere et al. that deletion of the core motif of one 21U RNA does not abrogate expression of neighboring 21U RNAs, although the species assessed were distant, separated by multiple 21U RNA loci, and encoded on both strands [35].

*C. elegans* 21U RNA loci, like the piRNA loci of mouse and fly [9], [10], [14], are genomically clustered. The overwhelming majority of 21U RNAs map to two large regions on chromosome IV, and GTTTC, the most highly conserved five nt of the core motif, occurs much more frequently on chromosome IV at these regions (4.0 *occ*urrences per *k*ilo*b*ase, occ/kb) than on chromosome IV outside these regions (0.4 occ/kb) or on other chromosomes (0.2 occ/kb). Furthermore, 21U RNAs encoded on chromosome IV are detected at much higher abundance (mean abundance: 148 RPM) than those encoded on other chromosomes (1 RPM) (Welch's *t*-test, p = 2.4e-269). These observations suggest the possibility of a positional requirement for expression of 21U RNA loci: a privileged genomic environment might contribute to the expression of 21U RNAs. To investigate the significance of 21U RNA genomic organization, we carried out rough mapping of the genomic insertion sites of several of the high-copy transgenic arrays. None of the integration loci mapped to chromosome IV (Table 1), indicating that these strains are not expressing 21UR-synth from the context of the 21U RNA genomic clusters. Yet the transgenic arrays themselves could represent 21U RNA-rich genomic microenvironments, much like the chromosome IV 21U RNA clusters. We therefore tested the true autonomy of the 21U RNA by using the MosSCI technique [47] to insert single-copy transgenes at a locus on chromosome IV not contained within the 21U RNA genomic clusters. Local 21U RNA concentration at the integration site is low, and no 21U RNAs are annotated as mapping to the homology arms encoded on the pCFJ178 MosSCI plasmid. Unexpectedly, single-copy insertions of ♂Tg1258 and ♀Tg2502 transgenes express 21UR-synth at levels easily detectable, albeit tenfold lower than the high-copy arrays. As observed for the high-copy arrays, scrambling of the core motif severely diminishes expression of 21UR-synth from the single-copy transgenic insertions (Figure 6F). Finally, to exclude the remote possibility that chromosome IV origin itself is essential for 21U RNA expression, we used an alternative MosSCI plasmid to insert onto chromosome II a single copy of the ♀Min2502 transgene, which encodes no other 21U RNAs. Like the chromosome IV transgene insertions, ♀Min2502 expresses 21UR-synth robustly (Figure 6F), confirming that 21U RNAs can be autonomously transcribed.

## Discussion

### piRNAs are transcribed as tiny, autonomous transcriptional units

Our data support a 21U RNA biogenesis mechanism wherein the upstream motif and 21U RNA sequence constitute a tiny, independent transcriptional unit that encodes regulated germline expression. The upstream motif as initially identified by Ruby et al. [12] is necessary for autonomous expression of a 21U RNA from one or more optimally situated downstream genomic thymidines. Importantly, this genomic thymidine may not represent a transcriptional requirement but rather reflect the binding preferences of the Argonaute PRG-1: a heterogeneous pool of candidate 21U RNA sequences may be transcribed and subsequently purified through preferential stabilization by PRG-1. Our transgenic studies showing greatly decreased expression when 21UR-synth is mutated to 21[U>A/G]R-synth cannot differentiate between a transcriptional or post-transcriptional requirement for a 5′ uridine; however, findings in other organisms support the latter mechanism. In mouse and fly, the prevailing model posits that Zucchini generates candidate primary piRNA 5′ ends with very little sequence specificity during the processing step, and then Piwi preferentially binds 5′ uridine piRNAs during the loading step [16], [17]. This is consistent with in vitro data showing that Siwi, the silkworm ortholog of PRG-1, preferentially incorporates ssRNAs bearing a 5′ uridine [18].

### On the evidence for transcription of 21U RNAs by RNA polymerase II

The upstream motif differences of male and female 21U RNAs suggest that germline enrichment could be achieved through selective transcription in male versus female germlines. Recently, Cecere et al. reported that 21U RNA upstream regions are depleted of nucleosomes [35]. They further observed that RNA polymerase II (Pol II) occupancy shows local peaks in this region, rising steadily over the interval of −300 nt to −50 nt from the genomic thymidine encoding the 5′ uridine of the 21U RNA. Analyzing the same ChIP-seq dataset as Cecere et al., we noticed that the amplitude of the changes in Pol II occupancy at 21U RNA loci is quite modest. Analyzing randomly generated intergenic windows from chromosome IV, we determined that the Pol II ChIP-seq background actually exceeds the “signal” at 21U RNA loci (Figure S7A, S7B), indicating relative Pol II depletion. This overall depletion of Pol II occupancy at 21U RNA loci may indicate that transcription of 21U RNAs is a more transient process than transcription of genes with canonical promoter elements. Thus the ChIP-seq might capture only a small fraction of interactions between Pol II and DNA. However, the Pol II occupancy profiles for the loci encoding the top 25% and bottom 25% of 21U RNAs by abundance are virtually indistinguishable (Figure S7C). Again, this is in stark contrast to mRNA coding loci, for which Pol II occupancy at the top 25% of mRNAs by abundance is much higher than at the bottom 25% (Figure S7D). An alternative possibility is that the open chromatin of the nucleosome-depleted regions upstream of 21U RNA loci is more susceptible to incidental binding by Pol II, causing the modest increase in local occupancy observed by Cecere et al. Should this be the case, the products of Pol II transcription at these loci could be unrelated to 21U RNAs. Cecere et al. also identify a transcript whose 5′ end extends 2 nt upstream of a 21U RNA locus and note that deep sequencing of 5′ capped RNAs reveals many more such transcripts. While these transcripts may represent 21U RNA precursors, they may also represent the products of incidental transcription from 21U RNA loci exposed due to local nucleosome depletion. The levels of such long putative precursors were below the threshold of our detection, precluding further study. Nevertheless, the uncertain 5′ nucleotide identity of the nascent 21U RNA transcript does not affect the interpretation of our results. Further studies, including identification of a cleavage mechanism for the 2 nt 5′ overhang, are needed to confirm these capped transcripts as bona fide 21U RNA precursors. The Zucchini endoribonuclease, thought to generate piRNA 5′ ends in mouse and fly [16], [17], is not a likely candidate, as it has no obvious homolog in *C. elegans* and shows very little sequence specificity, nor is there any evidence in *C. elegans* for processing of a long 21U RNA precursor into multiple species.

### How are the male and female subsets of 21U RNAs differentially expressed?

We show that the 5′ nucleotide of the conserved core motif influences germline enrichment of the dependent 21U RNA species (Figure 4, Figure 6). This differential expression of male and female 21U RNAs may be orchestrated by DNA-binding proteins that differ in germline expression patterns and/or binding affinity for 5′ cytidine core motifs. Recently, Cecere et al. demonstrated that the forkhead transcription factors UNC-130, FKH-3, and FKH-5 specifically bind a CTGTTTCA-containing substrate dsDNA probe in vitro [35]. However, male and female 21U RNAs do not appear to be differentially sensitive to depletion of these forkhead proteins, nor do 21U RNAs with and without 5′ cytidine motifs (data not shown and [35]). Cecere et al. propose that these forkhead proteins play a redundant role in transcription of 21U RNAs. While these are dispensable for viability and fertility, other forkhead proteins are required for development of the germline, precluding testing for a role in transcribing 21U RNAs; these additional forkhead proteins could indeed represent germline-specific or motif-specific transcription factors (Figure S8).

### Why are autonomous 21U RNA transcriptional units genomically clustered?

The autonomy of the *C. elegans* piRNA gene raises the questions of why 21U RNA loci exhibit genomic clustering on chromosome IV and why 21U RNAs encoded on chromosome IV are expressed more robustly. Perhaps the high density of 21U RNAs within these genomic clusters evolved as such: 21U RNA loci, defined by 21U RNA core motifs flanked by A/T richness, accumulated randomly on ancestral chromosome IV. Targeting of any overlapping genes resulted in silencing, subjecting the coding sequences of these genes to drift and eventual elimination. This would deplete the region of genes, reducing selection upon the genomic sequence and thereby permitting further accumulation of 21U RNA loci. The lack of selective pressure related to conservation of protein-coding genes might also explain why chromosome IV loci express 21U RNAs most robustly: the high density of coding and regulatory elements on other chromosomes likely constrains the evolution of features such as flanking A/T-richness that might enhance 21U RNA expression. It is also possible that different transcriptional machineries or different chromatin configurations are required to transcribe 21U RNAs versus other elements.

Genomic clustering of piRNA loci has been proposed to provide a “trap” for mobile elements [14]. In organisms such as mouse and fly where these clusters are transcribed to generate long precursors from which piRNAs are processed [9], [10], [14], the trapping function of the genomic piRNA cluster is readily apparent. Although the 21U RNAs are independently transcribed, Bagijn et al. have identified a similar mechanism acting in *C. elegans*: the genome shows evidence of recent transposon integration downstream of the conserved upstream 21U RNA motif, sometimes generating 21U RNAs that are antisense to the transposon 3′ end and capable of silencing it [29]. Each conserved upstream motif can therefore serve as an independent trap, with the result that increased accumulation of motifs enhances protection against mobile elements. While retroelements comprise over 40% of the human genome, they appear to have been strongly counterselected in *C. elegans*, where they constitute only 0.2% of the genome [52]. Perhaps the autonomous piRNA mechanism at play in *C. elegans* has rendered the animal less susceptible to this kind of mobile element over an evolutionary time scale. Intriguingly, however, *C. elegans* shows significantly higher rates of gene duplication than fly [53], and the *C. elegans* genome shows substantial expansions of gene families; for example, the *C. elegans* Argonaute family has expanded to over two dozen members, with the evolution of a worm-specific clade. As gene duplications, like mobile elements, may also be targeted by piRNAs, the preponderance of gene family expansions in *C. elegans* could suggest that this system confers enhanced protection against transposons at the expense of enhanced tolerance for gene duplications. Identification of additional organisms that use similar mechanisms for generating piRNAs will reveal whether this is a pattern or a peculiarity of *C. elegans*.

#### Note added in proof

Gu et al. recently identified global candidate RNA polymerase II transcription start sites by deep sequencing of capped RNAs [54]. For a large proportion of annotated 21U RNAs, the authors identified 5′ capped, ∼26 nt putative precursors with a 2 nt 5′ overhang. Longer RNA reads (70–90 nt) were identified overlapping a very small minority of 21U RNA loci. Abundance of these longer reads correlated poorly with 21U RNA abundance, while the abundance of the short, ∼26 nt reads correlated well, suggesting they are likelier to represent 21U RNA precursors. The 5′ cap structure of the putative 21U RNA precursor indeed suggests transcription by Pol II, although our analysis of Pol II occupancy data is inconclusive.

## Materials and Methods

### Strains

*C. elegans* were maintained according to standard procedures. The Bristol strain N2 was used as the standard wild-type strain. The alleles used in this study, listed by chromosome, are: unmapped: *xkIs11[♂Scram1258 cb-unc-119(+)]*, *xkIs12[♀Scram2502 cb-unc-119(+)]*, *xkIs14[♂21U>A1258 cb-unc-119(+)]*, *xkIs15[♂21U>G1258 cb-unc-119(+)]*, *xkIs16[♂Min1258 cb-unc-119(+)]*, *xkIs17[♀21U>A2502 cb-unc-119(+)]*, *xkIs18[♀21U>G2502 cb-unc-119(+)]*, *xkIs19[♀Min2502 cb-unc-119(+)]*, *xkIs20[♂Min1415 cb-unc-119(+)]*, *xkIs21[♂MinC>A1415 cb-unc-119(+)]*, *xkIs22[Tg1415-Tg2109 cb-unc-119(+)]*, *xkIs23[Scram1415-Tg2109 cb-unc-119(+)]*; LGX: *xkIs10[♀A>C2502 cb-unc-119(+)]*; LGI: *glp-4(bn2)*, *prg-1(tm872)*, *xkIs5[♀Tg2502 cb-unc-119(+)]*; LGII: *xkSi30 [♀Min2502 cb-unc-119(+)]*, *xkIs6[♂C>A1258 cb-unc-119(+)]*; LGIII: *rde-4(ne301), henn-1(tm4477)*; LGIV: *xkSi3[♂Tg1258 cb-unc-119(+)]*, *xkSi13[♀Tg2502 cb-unc-119(+)]*, *xkSi17[♂C>A1258 cb-unc-119(+)]*, *xkSi20[♀A>C2502 cb-unc-119(+)]*, *xkSi23[♂Scram1258 cb-unc-119(+)]*, *xkSi28[♀Scram2502 cb-unc-119(+)]*, *fem-1(hc17)*, *him-8(e1489)*; LGV: *fog-2(q71)*, *xkIs1[♂Tg1258 cb-unc-119(+)]*. Transgenic allele details and corresponding strain names are shown in Table 1.

### Sample collection and small RNA analysis

*C. elegans* samples were generated as previously described [24]. Samples for Taqman RT-qPCR validation of 21U RNA germline enrichment classification analysis were collected in biological duplicate. Samples collected for RNA-immunoprecipitation (RIP) analysis were collected in biological duplicate and analyzed in independent experiments with technical duplicates. All other samples were collected in biological triplicate. All samples analyzed represent adult animals unless otherwise stated.

RNA isolation, beta-elimination, northern blot analysis, Taqman RT-qPCR, and mRNA quantitation were performed as previously described [24]. RIP analysis was performed as follows: A custom rabbit polyclonal anti-PRG-1 antibody was generated by Proteintech Group, Inc using an N-terminal peptide antigen (MASGSGRGRGRGSGSNNS (C)) conjugated to keyhole limpet hemocyanin (KLH) carrier protein. Antisera were affinity purified using Affi-Gel 10 gel (Bio-Rad). PRG-1 was purified from synchronized gravid animals using this anti-PRG-1 rabbit polyclonal antibody. For each IP, 10 µg of anti-PRG-1 antibody was cross-linked to Dynabeads Protein A (Invitrogen) and incubated with lysate prepared from 0.3 ml of frozen worms at 4°C for 1 hr. Beads were washed 4× with RIP wash buffer (50 mM Tris-HCL pH 7.5, 200 mM KCL and 0.05% NP-40). After final wash, beads were split into equal volumes for RNA extraction and western blot procedure. For western blot analysis: 30 ul of 1× Tris-glycine SDS sample buffer (Invitrogen) without DTT was added directly to beads and incubated at 50°C for 10 min. 0.1 M DTT was then added to samples and boiled for 5 min before loading on gel. Proteins immobilized on Immobilon-FL transfer membrane (Millipore) were probed with anti-PRG-1 rabbit polyclonal antibody or anti-gamma-tubulin rabbit polyclonal antibody (LL-17) (Sigma) (1∶2,000). Peroxidase-AffiniPure goat anti-rabbit IgG secondary antibody was used at 1∶10,000 (Jackson ImmunoResearch Laboratories) for detection using Pierce ECL Western Blotting Substrate (Thermo Scientific). For RNA extraction: 1 ml of TRI-Reagent (Ambion) was directly added to beads and incubated at room temperature for 5 min. RNAs were precipitated in isopropanol for 1 hr at −30°C followed by three washes with 70% ethanol.

Small RNA quantitation was performed as previously described [24]. All 21U RNA qPCR data from transgenic studies were normalized to miR-1 levels. As a result of this normalization, some small RNAs whose levels are not detectable (cycle number >36) appear to be detected due to small variation in detection of miR-1. 21UR-synth is not detectable in non-transgenic animals at any stage at which it was assessed. All *Cbr-unc-119* qPCR data were normalized to *act-1* mRNA levels. The sequence of 21UR-synth is 5′ TGATATGCGATGTAGTAGACT 3′. The sequence of 21UR-synthB is 5′ TTAGTCGTATGTGACGCTGCC 3′. Full small RNA sequences were submitted to Applied Biosystems for design of Taqman assays. Northern blot probe sequences used for this study: miR-1 5′ TACATACTTCTTTACATTCCA /3StarFire/ 3′; ♀21UR-2502 5′ CAGCAGTCTACTACAATTTCA /3StarFire/ 3′; 21UR-synth 5′ AGTCTACTACATCGCATATCA /3StarFire/ 3′. RT-qPCR primer sequences used for this study are as follows: *act-1* F 5′ CCAGGAATTGCTGATCGTATGCAGAA 3′, R 5′ TGGAGAGGGAAGCGAGGATAGA 3′; *Cbr-unc-119* F 5′ AACGACGTTTTAGCACTTCCG 3′, R 5′ GGATTTGGAACTTGGTGAACTCG 3′.

### *C. elegans* transgenesis

To generate the base of the 1258 transgene, sequence spanning genomic coordinates IV:14390835–14393692 was used; IV:14392513–14392673 was used for the *♂*Min1258 transgene. To generate the base of the 2502 transgene, sequence spanning genomic coordinates IV:15395699–15397722 was used; IV:15396667–15396886 was used for the ♀Min2502 transgene. To generate the base of the Min1415 transgene, sequence spanning genomic coordinates IV:16564187–16564395 was used. To generate the base of the Tg1415–Tg2109 transgene, sequence spanning genomic coordinates IV:16564133–16564395 was used; the Tg1415–Tg2109 and Scram1415-Tg2109 transgenes carry a 13 nt deletion downstream of both 21U RNA loci. Coordinates were taken from the *C. elegans* genome WS220. The mutations described in Table 1 were introduced through site-directed mutagenesis or inverse PCR with phosphorylated primers. Transgenes were then subcloned into the pCFJ178 (IV) or pCFJ151 (II) vector. The chromosome IV transgene insertion site lies outside the larger 21U RNA genomic clusters, and the homology arms of chromosome IV MosSCI vector pCFJ178 do not encode any annotated 21U RNAs. Transgenes were confirmed by sequencing and injected into animals with pharyngeal and/or body wall muscle coinjection markers to distinguish transgenic animals. High-copy arrays were integrated through ultraviolet irradiation. MosSCI single-copy insertions were generated as previously described [47].

### Small RNA sequencing data acquisition and linker removal

Raw data files from 24 small RNA sequencing experiments [27], [36]–[42] were downloaded from NCBI Gene Expression Omnibus [55]. Artificial linker sequences were removed using an in-house linker removal pipeline. We first searched each sequence for a perfect match to the linker. If a perfect match was not found, we searched for an alignment to the linker with 1 mismatch. If not found, we searched for a perfect alignment between the last 5 nt of the sequence and the first 5 nt of the linker. If not found, we repeated this search allowing 1 mismatch. We continued this pattern to align 4 and 3 nt. Sequences with no linker alignment were discarded (∼20% of reads).

### Small RNA read alignment to genome and annotation to 21U RNAs

Reads were aligned to the reference *C. elegans* genome version WS220 using Bowtie [56] with the following parameters: -f -v 2 -k 50 --best --strata. Mapped read counts in each library were normalized to the number of total mapped reads in that library and to the number of mapped genomic loci. Sequence abundance is reported as reads per million mapped reads (RPM). To determine 21U RNA abundance, we first generated 21U RNA genomic coordinates by aligning 15,703 known 21U RNA sequences [27] to the *C. elegans* genome version WS220 using Bowtie. Perfect, full-length alignments for 15,093 of these sequences were considered valid 21U RNA coordinates. Reads mapping entirely within these coordinates were annotated to 21U RNAs.

### Enrichment Score calculations

Germline enrichment classifications of 21U RNAs were generated based on read counts in 17 germline libraries: 14 male germline libraries prepared from isolated spermatogenic cells, isolated spermatids, or whole adult males; and 3 female germline libraries prepared from purified oocytes or whole adult hermaphrodites defective in sperm production (Table S1). 1,198 21U RNAs had no read counts in any of these libraries and were removed from our analysis. 184 21U RNAs had higher read counts in a *prg-1(tm872)* young adult library compared to an N2 young adult library [27] and were removed from our analysis, leaving 13,711 21U RNAs for which we assessed germline enrichment. Libraries generated using a 5′-monophosphate-dependent (5 male, 1 female) versus -independent (9 male, 2 female) protocol were separated for calculation of the Enrichment Score as follows: For each 21U RNA, we calculated fold abundance difference between every male and female library, for a total of 23 comparisons. Each 21U RNA began with an Enrichment Score of 0. For every comparison, if the 21U RNA was more then 5-fold abundant in the male library, the Enrichment Score decreased by 1; if the 21U RNA was more than 5-fold abundant in the female library, the Enrichment Score increased by 1. Male 21U RNAs were defined as those with Enrichment Scores ≤−3, while female 21U RNAs were defined as those with Enrichment Scores ≥3. Remaining 21U RNAs were classified as non-enriched. To validate enrichment classifications, the fold abundance differences for each 21U RNA were averaged across all 23 comparisons. Less than 1% of 21U RNAs classified as male or female do not show enrichment by average fold abundance in their respective libraries. These 21U RNAs were reclassified as non-enriched for subsequent analyses. 21U RNA Enrichment scores and germline enrichment classifications are in Dataset S1.

### Determination of false discovery rate

To approximate the number of 21U RNAs falsely classified as male or female germline-enriched by our method, we performed Enrichment Score calculations on randomly generated count data modeled from an N2 young adult library [27]. 11,458 21U RNAs are represented in this library. Because 17 germline libraries were used for the real analysis, we generated 17 control libraries as follows: For each 21U RNA, 17 random counts were generated from a Poisson distribution with λ = α (where α is set to the 21U RNA count in the N2 library) and assigned to one of 17 control libraries. After all counts were assigned, the 17 control libraries were randomly grouped to represent the number of male or female and 5′-monophosphate-dependent or -independent libraries used above. Enrichment Score calculations were then performed on these control libraries as described above, and the number of 21U RNAs classified as germline-enriched was calculated. This protocol was repeated 1,000 times. On the basis of this randomized data, we defined an Enrichment Score threshold of −/+3, inclusive, for classifying 21U RNAs as male or female germline-enriched, respectively. Application of this threshold to the randomized data resulted in classification of, on average, only 0.76% (101 of 11,458) of 21U RNAs as germline-enriched, corresponding to a false discovery rate below 1%. This value is consistent with the less than 1% of 21U RNAs classified as male or female that do not show enrichment by average fold abundance in their respective libraries.

### Enrichment Score calculations performed on 26G RNAs

26G RNA annotations were taken from Han et al., 2009 [38]. The abundances of 4,002 26G RNAs were measured in 13 of the 17 libraries used for 21U RNA Enrichment Score calculations. Four male libraries (GSM465244, GSM503843, GSM459329, and GSM459331) were excluded because the animals used in preparation of the libraries carried mutations in genes required for 26G RNA expression [36], [40], [41]. Enrichment Score calculations were performed on the 13 remaining libraries as above, for a total of 16 male∶female comparisons. We retained the Enrichment Score threshold for classifying 26G RNAs as male or female germline-enriched.

### Analysis of 21U RNA–dependent 22G RNAs and 21U RNA targets

21U RNA target and 22G RNA information for young adult animals (N2 and *prg-1(n4357)*) was obtained from Bagijn et al. [29]; raw sequencing data files for gravid adult animals (N2 and *prg-1(n4357)*) were downloaded from GEO [30]. Raw sequences were processed as described above, and reads 22 nt long and starting with guanosine were annotated as 22G RNAs. 21U RNA targets were defined as transcripts with 0–3 mismatches to a 21U RNA sequence. 21U RNA-dependent 22G RNAs were defined as 22G RNAs that map antisense to transcripts within 40 nt of a 21U RNA target site. The number of 22G RNAs that map to both male and female 21U RNA target sites was compared to a control number of 22G RNAs that map to both a random set of male and a random set of female 21U RNA target sites. These random target sites were defined as the target sites of 7,677 randomly selected 21U RNAs representing “male” 21U RNAs and the target sites of 2,171 randomly selected (and not overlapping random male) 21U RNAs to represent “female” 21U RNAs. This random selection was repeated 1,000 times. A similar randomization process was repeated to compare with the number of genes targeted by both male and female 21U RNAs.

### Core motif visualization

Core motifs of 21U RNAs were visualized using WebLogo and correcting for *C. elegans* genome nucleotide composition [57]. To account for variability in the location of core motifs relative to their 21U RNA loci, upstream regions were aligned by the central 3 Ts of the core motif. If no core motif was identified within 60 nt upstream of a 21U RNA, we aligned position −44 relative to the 21U RNA locus to the G of the core motif, corresponding to the previously identified most common position of the G [12]. Only 21U RNAs that map to a single locus in the genome (13,387 of 13,711 21U RNAs, 97.6%) were analyzed since 21U RNAs that map to more than 1 locus may have different upstream sequences.

### Identification of genomic features for nucleosome and Pol II occupancy profiling

Nucleosome and Pol II occupancy profiling for 21U RNA loci was centered on the genomic thymidine encoding the 21U RNA 5′ uridine. Profiling for transcripts was centered on transcription start sites (TSS) defined as the start of 5′ UTRs annotated in the Ensembl66 database [58]. Intergenic regions were defined as regions absent of an annotated 5′ UTR, exon, intron, 3′ UTR or small RNA transcript that were partitioned into randomly distributed, non-overlapping 1,000 nt windows. Profiling for intergenic regions was centered on these 1,000 nt windows.

Young adult TSS expression was calculated as fragments per kilobase per million mapped reads (FPKM) using biological replicates from a transcriptomic sequencing experiment [59]. Transcriptome sequence data were removed of linkers and aligned to the *C. elegans* genome version WS220 using TopHat [60]. Cufflinks [61] was used to calculate transcript isoform expression. Transcripts with an annotated 5′ UTR were extracted from the Ensembl66 database. Average transcript FPKM across the two libraries was calculated, and the isoform with the highest expression was chosen for nucleosome and Pol II occupancy analyses. For isoforms with equivalent expression, a single isoform was randomly chosen.

### Analysis of nucleosome and Pol II occupancy

Published nucleosome occupancy data [62] were downloaded from UCSC, and the genomic coordinates were lifted over from WS170 to WS220. Adjusted nucleosome occupancy data centered on 21U RNAs, TSS, and an intergenic background control were averaged for each nucleotide. Pol II ChIP-seq data from young adult worms were downloaded from the modEncode repository [63]. Pol II signal to input ratios on chromosome IV were averaged for each nucleotide. TSS were further filtered to only include transcripts with at least 5 FPKM as calculated above.

Pol II ChIP-seq data from young adult worms were downloaded from the modEncode repository. Pol II signal to input ratios were averaged for each nucleotide separately for male and female 21U RNAs on chromosome IV.

## Supporting Information

Dataset S1 List of 21U RNAs analyzed in this study, their Enrichment scores, their germline enrichment classifications based on Enrichment scores (Classification by score), and final germline enrichment classifications after removal of 21U RNAs whose average fold abundance in the enriched germline was not higher than the non-enriched germline (Adj. for avg. fold abundance).
(XLSX)

Figure S1 Computational identification of male and female germline-enriched 21U RNAs. (A) Enrichment Score calculations performed on 17 small RNA sequencing libraries classify a majority of 21U RNAs as male (blue) or female (red) germline-enriched. Non-enriched (NE) 21U RNAs, grey. Numbers indicate percent of 13,711 21U RNAs analyzed. (B) Enrichment Score calculations performed on control data classify <1% of 21U RNAs as male or female germline-enriched indicating a 1% false discovery rate. Numbers indicate percent of 11,458 21U RNAs analyzed. (C) Male 21U RNAs are more abundant in male libraries. Average relative abundance of each male 21U RNA was calculated between each of the 23 male∶female library comparisons. (D) Female 21U RNAs are more abundant in female libraries. Average relative abundance of each male 21U RNA was calculated between each of the 23 female∶male library comparisons. (E) Non-enriched 21U RNAs are equally abundant in male and female libraries.
(PDF)

Figure S2 Female 21U RNAs are preferentially abundant in embryo. (A) Relative male 21U RNA abundance is decreased in embryo. Average relative abundance of each male 21U RNA was calculated between each of 5 male and 4 mixed stage embryo libraries. Dotted line indicates equal male and embryo abundance. Pie chart depicts proportion of male 21U RNAs with reads in at least one embryo library (dark blue). (B) A population of female 21U RNAs shows increased abundance in embryo. Average relative abundance of each female 21U RNA was calculated between each of 1 female and 4 mixed stage embryo libraries. Pie chart depicts proportion of female 21U RNAs with reads in at least one embryo library (dark red). (C,D) Taqman RT-qPCR analysis corroborates male 21U RNA depletion in embryo. Expression of representative male 21U RNAs was assayed by Taqman in *him-8(e1489)* (C) and *fog-2(q71)* (D) male animals and N2 embryos. Error bars represent ±1 SD from two biological replicates. (E) Taqman RT-qPCR analysis corroborates female 21U RNA enrichment in embryo. Expression of representative female 21U RNAs was assayed by Taqman in *fem-1(hc17)* female animals and N2 embryos. (F) Male germline-enriched 26G RNAs are generally absent in embryo. Average relative abundance of each male 26G RNA was calculated between each of 4 male and 4 mixed stage embryo libraries. (G) Female germline-enriched 26G RNAs are robustly expressed in embryo. Average relative abundance of each female 26G RNA was calculated between each of 1 female and 4 mixed stage embryo libraries.
(PDF)

Figure S3 21U RNAs target significantly non-overlapping sets of genes. (A) 22G RNAs are almost exclusively derived from either male or female 21U RNAs, but not both. The number of unique 22G RNAs derived from both male and female 21U RNAs is significantly less than expected if 22G RNAs are selected at random (Fisher's exact test, p = 1.2e−02). (B) Male and female 21U RNAs target significantly fewer overlapping genes compared to selecting random sets of genes (Fisher's exact test, p = 7.7e−13). (C) 5,956 genes targeted by male 21U RNAs in young adult (YAd) animals are depleted of spermatogenesis genes compared to a random set of 5,956 genes. (D) 1,387 genes targeted by female 21U RNAs in gravid adult (GA) animals are enriched for oogenesis genes compared to a random set of 1,387 genes.
(PDF)

Figure S4 Transgenic array expression varies across transgenes. (A) Levels of *Cbr-unc-119* mRNA in adult animals were assayed by RT-qPCR for all transgenes and normalized to *act-1* mRNA levels. (B) Expression of transgenic 21UR-synth does not affect expression of endogenous 21U RNA counterparts. Endogenous ♂21UR-1258 and ♀21UR-2502 levels were assayed by Taqman RT-qPCR and normalized to microRNA miR-1 levels in all samples.
(PDF)

Figure S5 21U RNAs are specifically immunoprecipitated with PRG-1 complexes. (A) anti-PRG-1 antibody does not immunprecipitate microRNA miR-1. (B) 21UR-synth expression does not interfere with association of endogenous 21U RNAs with PRG-1.
(PDF)

Figure S6 21U RNA expression requires a 5′ genomic thymidine. (A) Schematic of transgenes encoding 21UR-synth with different 5′ nt. (B–C) Mutation of the 5′ genomic thymidine disrupts expression of 21UR-synth by northern blot (B) and Taqman assay (C). WT and ♀Tg2502 lanes in (B) are repeated from Figure 5B for clarity.
(PDF)

Figure S7 RNA polymerase II occupancy at 21U RNA loci is below background level. (A) Average Pol II occupancy in a young adult library of 21U RNA loci expressing 21U RNAs with at least 5 RPM (red), transcriptional start sites (TSS) expressing transcripts with at least 5 FPKM (green), and randomized intergenic regions (yellow). Only regions on ChrIV were assayed (B) Pol II occupancy as described in (A) but independently scaled for each transcript type and plotted with average nucleosome occupancy (black line). Grey error bands: SEM. (C) Average Pol II occupancy of 21U RNA loci as (B) but showing the top 25% 21U RNAs by abundance (1^st^ quartile) and the bottom 25% (4^th^ quartile) separately. 21U RNAs on all chromosomes are shown. (D) Same as (E) but showing top and bottom 25% of TSS by transcript abundance.
(PDF)

Figure S8 Model of 21U RNA expression.
(PDF)

Table S1 Descriptions of small RNA sequencing libraries used in this study. GEO Accessions for datasets and libraries used are listed. Libraries generated using 5′-monophosphate-dependent (Dep) or -independent (Indep) RNA extraction protocols are indicated along with how the library was used in this study (“Use” column).
(PDF)

Table S2 Welch's *t*-test p-values for all abundance comparisons between 21U RNAs with different core motifs. Highlighted are p-values <0.01. Identity of the 5′ nt corresponding to higher 21U RNA abundance is indicated below each significant p-value. All *t*-tests are two-tailed. Comparisons of abundances in 5′-monophosphate-dependent and -independent libraries were performed separately.
(PDF)

## References

[1] Grimson A, Srivastava M, Fahey B, Woodcroft BJ, Chiang HR, et al (2008) Early origins and evolution of microRNAs and Piwi-interacting RNAs in animals. Nature 455: 1193–1197. 18830242 10.1038/nature07415 PMC3837422

[2] Lin H, Spradling AC (1997) A novel group of pumilio mutations affects the asymmetric division of germline stem cells in the Drosophila ovary. Development 124: 2463–2476. 9199372 10.1242/dev.124.12.2463

[3] Cox DN, Chao A, Baker J, Chang L, Qiao D, et al (1998) A novel class of evolutionarily conserved genes defined by piwi are essential for stem cell self-renewal. Genes Dev 12: 3715–3727. 9851978 10.1101/gad.12.23.3715 PMC317255

[4] Schmidt A, Palumbo G, Bozzetti MP, Tritto P, Pimpinelli S, et al (1999) Genetic and molecular characterization of sting, a gene involved in crystal formation and meiotic drive in the male germ line of Drosophila melanogaster. Genetics 151: 749–760. 9927466 10.1093/genetics/151.2.749 PMC1460476

[5] Mochizuki K, Fine NA, Fujisawa T, Gorovsky MA (2002) Analysis of a piwi-related gene implicates small RNAs in genome rearrangement in tetrahymena. Cell 110: 689–699. 12297043 10.1016/s0092-8674(02)00909-1

[6] Carmell MA, Girard A, van de Kant HJ, Bourc'his D, Bestor TH, et al (2007) MIWI2 is essential for spermatogenesis and repression of transposons in the mouse male germline. Developmental cell 12: 503–514. 17395546 10.1016/j.devcel.2007.03.001

[7] Kuramochi-Miyagawa S, Watanabe T, Gotoh K, Totoki Y, Toyoda A, et al (2008) DNA methylation of retrotransposon genes is regulated by Piwi family members MILI and MIWI2 in murine fetal testes. Genes & Development 22: 908–917. 18381894 10.1101/gad.1640708 PMC2279202

[8] Li C, Vagin VV, Lee S, Xu J, Ma S, et al (2009) Collapse of germline piRNAs in the absence of Argonaute3 reveals somatic piRNAs in flies. Cell 137: 509–521. 19395009 10.1016/j.cell.2009.04.027 PMC2768572

[9] Aravin A, Gaidatzis D, Pfeffer S, Lagos-Quintana M, Landgraf P, et al (2006) A novel class of small RNAs bind to MILI protein in mouse testes. Nature 442: 203–207. 16751777 10.1038/nature04916

[10] Girard A, Sachidanandam R, Hannon GJ, Carmell MA (2006) A germline-specific class of small RNAs binds mammalian Piwi proteins. Nature 442: 199–202. 16751776 10.1038/nature04917

[11] Lau NC, Seto AG, Kim J, Kuramochi-Miyagawa S, Nakano T, et al (2006) Characterization of the piRNA complex from rat testes. Science 313: 363–367. 16778019 10.1126/science.1130164

[12] Ruby JG, Jan C, Player C, Axtell MJ, Lee W, et al (2006) Large-scale sequencing reveals 21U-RNAs and additional microRNAs and endogenous siRNAs in C. elegans. Cell 127: 1193–1207. 17174894 10.1016/j.cell.2006.10.040

[13] de Wit E, Linsen SE, Cuppen E, Berezikov E (2009) Repertoire and evolution of miRNA genes in four divergent nematode species. Genome Res 19: 2064–2074. 19755563 10.1101/gr.093781.109 PMC2775598

[14] Brennecke J, Aravin AA, Stark A, Dus M, Kellis M, et al (2007) Discrete small RNA-generating loci as master regulators of transposon activity in Drosophila. Cell 128: 1089–1103. 17346786 10.1016/j.cell.2007.01.043

[15] Gunawardane LS, Saito K, Nishida KM, Miyoshi K, Kawamura Y, et al (2007) A slicer-mediated mechanism for repeat-associated siRNA 5′ end formation in Drosophila. Science 315: 1587–1590. 17322028 10.1126/science.1140494

[16] Ipsaro JJ, Haase AD, Knott SR, Joshua-Tor L, Hannon GJ (2012) The structural biochemistry of Zucchini implicates it as a nuclease in piRNA biogenesis. Nature 491: 279–283. 23064227 10.1038/nature11502 PMC3493678

[17] Nishimasu H, Ishizu H, Saito K, Fukuhara S, Kamatani MK, et al (2012) Structure and function of Zucchini endoribonuclease in piRNA biogenesis. Nature 491: 284–287. 23064230 10.1038/nature11509

[18] Kawaoka S, Izumi N, Katsuma S, Tomari Y (2011) 3′ end formation of PIWI-interacting RNAs in vitro. Mol Cell 43: 1015–1022. 21925389 10.1016/j.molcel.2011.07.029

[19] Vagin VV, Sigova A, Li C, Seitz H, Gvozdev V, et al (2006) A distinct small RNA pathway silences selfish genetic elements in the germline. Science 313: 320–324. 16809489 10.1126/science.1129333

[20] Kirino Y, Mourelatos Z (2007) Mouse Piwi-interacting RNAs are 2′-O-methylated at their 3′ termini. Nature Structural & Molecular Biology 14: 347–348. 10.1038/nsmb1218 17384647

[21] Ohara T, Sakaguchi Y, Suzuki T, Ueda H, Miyauchi K (2007) The 3′ termini of mouse Piwi-interacting RNAs are 2′-O-methylated. Nature Structural & Molecular Biology 14: 349–350. 10.1038/nsmb1220 17384646

[22] Kurth HM, Mochizuki K (2009) 2′-O-methylation stabilizes Piwi-associated small RNAs and ensures DNA elimination in Tetrahymena. RNA 15: 675–685. 19240163 10.1261/rna.1455509 PMC2661841

[23] Houwing S, Kamminga LM, Berezikov E, Cronembold D, Girard A, et al (2007) A role for Piwi and piRNAs in germ cell maintenance and transposon silencing in Zebrafish. Cell 129: 69–82. 17418787 10.1016/j.cell.2007.03.026

[24] Billi AC, Alessi AF, Khivansara V, Han T, Freeberg M, et al (2012) The Caenorhabditis elegans HEN1 Ortholog, HENN-1, Methylates and Stabilizes Select Subclasses of Germline Small RNAs. PLoS Genet 8: e1002617 doi:10.1371/journal.pgen.1002617. 22548001 10.1371/journal.pgen.1002617 PMC3330095

[25] Montgomery TA, Rim Y-S, Zhang C, Dowen RH, Phillips CM, et al (2012) PIWI Associated siRNAs and piRNAs Specifically Require the Caenorhabditis elegans HEN1 Ortholog henn-1. PLoS Genet 8: e1002616 doi:10.1371/journal.pgen.1002616. 22536158 10.1371/journal.pgen.1002616 PMC3334881

[26] Kamminga L, van Wolfswinkel J, Luteijn M, Kaaij L, Bagijn M, et al (2012) Differential impact of the Hen1 homolog HENN-1 on 21U and 26G RNAs in the germline of Caenorhabditis elegans. PLoS Genet 8: e1002702 doi:10.1371/journal.pgen.1002702. 22829772 10.1371/journal.pgen.1002702 PMC3400576

[27] Batista PJ, Ruby JG, Claycomb JM, Chiang R, Fahlgren N, et al (2008) PRG-1 and 21U-RNAs interact to form the piRNA complex required for fertility in C. elegans. Molecular Cell 31: 67–78. 18571452 10.1016/j.molcel.2008.06.002 PMC2570341

[28] Das PP, Bagijn MP, Goldstein LD, Woolford JR, Lehrbach NJ, et al (2008) Piwi and piRNAs act upstream of an endogenous siRNA pathway to suppress Tc3 transposon mobility in the Caenorhabditis elegans germline. Molecular Cell 31: 79–90. 18571451 10.1016/j.molcel.2008.06.003 PMC3353317

[29] Bagijn MP, Goldstein LD, Sapetschnig A, Weick EM, Bouasker S, et al (2012) Function, Targets, and Evolution of Caenorhabditis elegans piRNAs. Science 337: 574–8. 22700655 10.1126/science.1220952 PMC3951736

[30] Lee HC, Gu W, Shirayama M, Youngman E, Conte D Jr, et al (2012) C. elegans piRNAs Mediate the Genome-wide Surveillance of Germline Transcripts. Cell 150: 78–87. 22738724 10.1016/j.cell.2012.06.016 PMC3410639

[31] Billi AC, Freeberg MA, Kim JK (2012) piRNAs and siRNAs collaborate in Caenorhabditis elegans genome defense. Genome Biology 13: 164. 22818087 10.1186/gb-2012-13-7-164 PMC3491375

[32] Shirayama M, Seth M, Lee HC, Gu W, Ishidate T, et al (2012) piRNAs initiate an epigenetic memory of nonself RNA in the C. elegans germline. Cell 150: 65–77. 22738726 10.1016/j.cell.2012.06.015 PMC3597741

[33] Ashe A, Sapetschnig A, Weick EM, Mitchell J, Bagijn MP, et al (2012) piRNAs can trigger a multigenerational epigenetic memory in the germline of C. elegans. Cell 150: 88–99. 22738725 10.1016/j.cell.2012.06.018 PMC3464430

[34] Luteijn MJ, van Bergeijk P, Kaaij LJ, Almeida MV, Roovers EF, et al (2012) Extremely stable Piwi-induced gene silencing in Caenorhabditis elegans. The EMBO journal 31: 3422–3430. 22850670 10.1038/emboj.2012.213 PMC3419935

[35] Cecere G, Zheng GX, Mansisidor AR, Klymko KE, Grishok A (2012) Promoters recognized by forkhead proteins exist for individual 21U-RNAs. Molecular Cell 47: 734–745. 22819322 10.1016/j.molcel.2012.06.021 PMC3444671

[36] Gent JI, Schvarzstein M, Villeneuve AM, Gu SG, Jantsch V, et al (2009) A Caenorhabditis elegans RNA-directed RNA polymerase in sperm development and endogenous RNA interference. Genetics 183: 1297–1314. 19805814 10.1534/genetics.109.109686 PMC2787422

[37] Gu W, Shirayama M, Conte D, Vasale J, Batista PJ, et al (2009) Distinct argonaute-mediated 22G-RNA pathways direct genome surveillance in the C. elegans germline. Molecular Cell 36: 231–244. 19800275 10.1016/j.molcel.2009.09.020 PMC2776052

[38] Han T, Manoharan AP, Harkins TT, Bouffard P, Fitzpatrick C, et al (2009) 26G endo-siRNAs regulate spermatogenic and zygotic gene expression in Caenorhabditis elegans. Proc Natl Acad Sci USA 106: 18674–18679. 19846761 10.1073/pnas.0906378106 PMC2765456

[39] Kato M, de Lencastre A, Pincus Z, Slack FJ (2009) Dynamic expression of small non-coding RNAs, including novel microRNAs and piRNAs/21U-RNAs, during Caenorhabditis elegans development. Genome Biology 10: R54. 19460142 10.1186/gb-2009-10-5-r54 PMC2718520

[40] Gent JI, Lamm AT, Pavelec DM, Maniar JM, Parameswaran P, et al (2010) Distinct phases of siRNA synthesis in an endogenous RNAi pathway in C. elegans soma. Molecular Cell 37: 679–689. 20116306 10.1016/j.molcel.2010.01.012 PMC2838994

[41] Conine CC, Batista PJ, Gu W, Claycomb JM, Chaves DA, et al (2010) Argonautes ALG-3 and ALG-4 are required for spermatogenesis-specific 26G-RNAs and thermotolerant sperm in Caenorhabditis elegans. Proc Natl Acad Sci USA 107: 3588–3593. 20133686 10.1073/pnas.0911685107 PMC2840486

[42] Stoeckius M, Maaskola J, Colombo T, Rahn HP, Friedlander MR, et al (2009) Large-scale sorting of C. elegans embryos reveals the dynamics of small RNA expression. Nature methods 6: 745–751. 19734907 10.1038/nmeth.1370 PMC2756031

[43] Brennecke J, Malone CD, Aravin AA, Sachidanandam R, Stark A, et al (2008) An epigenetic role for maternally inherited piRNAs in transposon silencing. Science 322: 1387–1392. 19039138 10.1126/science.1165171 PMC2805124

[44] Chambeyron S, Popkova A, Payen-Groschene G, Brun C, Laouini D, et al (2008) piRNA-mediated nuclear accumulation of retrotransposon transcripts in the Drosophila female germline. Proceedings of the National Academy of Sciences of the United States of America 105: 14964–14969. 18809914 10.1073/pnas.0805943105 PMC2567476

[45] Grentzinger T, Armenise C, Brun C, Mugat B, Serrano V, et al (2012) piRNA-mediated transgenerational inheritance of an acquired trait. Genome Research 10.1101/gr.136614.111 PMC3460183 22555593

[46] Reinke V, Gil IS, Ward S, Kazmer K (2004) Genome-wide germline-enriched and sex-biased expression profiles in Caenorhabditis elegans. Development 131: 311–323. 14668411 10.1242/dev.00914

[47] Frokjaer-Jensen C, Davis MW, Hopkins CE, Newman BJ, Thummel JM, et al (2008) Single-copy insertion of transgenes in Caenorhabditis elegans. Nature genetics 40: 1375–1383. 18953339 10.1038/ng.248 PMC2749959

[48] Nolan T, Hands RE, Bustin SA (2006) Quantification of mRNA using real-time RT-PCR. Nature protocols 1: 1559–1582. 17406449 10.1038/nprot.2006.236

[49] Tabara H, Yigit E, Siomi H, Mello CC (2002) The dsRNA binding protein RDE-4 interacts with RDE-1, DCR-1, and a DExH-box helicase to direct RNAi in C. elegans. Cell 109: 861–871. 12110183 10.1016/s0092-8674(02)00793-6

[50] Sijen T, Steiner FA, Thijssen KL, Plasterk RH (2007) Secondary siRNAs result from unprimed RNA synthesis and form a distinct class. Science 315: 244–247. 17158288 10.1126/science.1136699

[51] Vasale JJ, Gu W, Thivierge C, Batista PJ, Claycomb JM, et al (2010) Sequential rounds of RNA-dependent RNA transcription drive endogenous small-RNA biogenesis in the ERGO-1/Argonaute pathway. Proceedings of the National Academy of Sciences of the United States of America 107: 3582–3587. 20133583 10.1073/pnas.0911908107 PMC2840456

[52] Bessereau JL (2006) Transposons in C. elegans. WormBook : the online review of C elegans biology 1–13. 18023126 10.1895/wormbook.1.70.1 PMC4781069

[53] Lynch M, Conery JS (2000) The evolutionary fate and consequences of duplicate genes. Science 290: 1151–1155. 11073452 10.1126/science.290.5494.1151

[54] Gu W, Lee HC, Chaves D, Youngman EM, Pazour GJ, et al (2012) CapSeq and CIP-TAP Identify Pol II Start Sites and Reveal Capped Small RNAs as C. elegans piRNA Precursors. Cell 151: 1488–1500. 23260138 10.1016/j.cell.2012.11.023 PMC3581324

[55] Barrett T, Troup DB, Wilhite SE, Ledoux P, Evangelista C, et al (2011) NCBI GEO: archive for functional genomics data sets–10 years on. Nucleic acids research 39: D1005–1010. 21097893 10.1093/nar/gkq1184 PMC3013736

[56] Langmead B, Trapnell C, Pop M, Salzberg SL (2009) Ultrafast and memory-efficient alignment of short DNA sequences to the human genome. Genome Biol 10: R25. 19261174 10.1186/gb-2009-10-3-r25 PMC2690996

[57] Crooks GE, Hon G, Chandonia J-M, Brenner SE (2004) WebLogo: a sequence logo generator. Genome Research 14: 1188–1190. 15173120 10.1101/gr.849004 PMC419797

[58] Flicek P, Amode MR, Barrell D, Beal K, Brent S, et al (2012) Ensembl 2012. Nucleic acids research 40: D84–90. 22086963 10.1093/nar/gkr991 PMC3245178

[59] Hillier LW, Reinke V, Green P, Hirst M, Marra MA, et al (2009) Massively parallel sequencing of the polyadenylated transcriptome of C. elegans. Genome Research 19: 657–666. 19181841 10.1101/gr.088112.108 PMC2665784

[60] Trapnell C, Pachter L, Salzberg SL (2009) TopHat: discovering splice junctions with RNA-Seq. Bioinformatics 25: 1105–1111. 19289445 10.1093/bioinformatics/btp120 PMC2672628

[61] Trapnell C, Williams BA, Pertea G, Mortazavi A, Kwan G, et al (2010) Transcript assembly and quantification by RNA-Seq reveals unannotated transcripts and isoform switching during cell differentiation. Nature biotechnology 28: 511–515. 10.1038/nbt.1621 PMC3146043 20436464

[62] Valouev A, Ichikawa J, Tonthat T, Stuart J, Ranade S, et al (2008) A high-resolution, nucleosome position map of C. elegans reveals a lack of universal sequence-dictated positioning. Genome Research 18: 1051–1063. 18477713 10.1101/gr.076463.108 PMC2493394

[63] Celniker SE, Dillon LA, Gerstein MB, Gunsalus KC, Henikoff S, et al (2009) Unlocking the secrets of the genome. Nature 459: 927–930. 19536255 10.1038/459927a PMC2843545

