# Supplementary figures and images for: A Conserved Upstream Motif Orchestrates Autonomous, Germline-Enriched Expression of Caenorhabditis elegans piRNAs
Source: PLoS Genet. 2013 Mar 14;9(3):e1003392. doi: 10.1371/journal.pgen.1003392 (PMC3597512; doi:10.1371/journal.pgen.1003392)

Figure S1

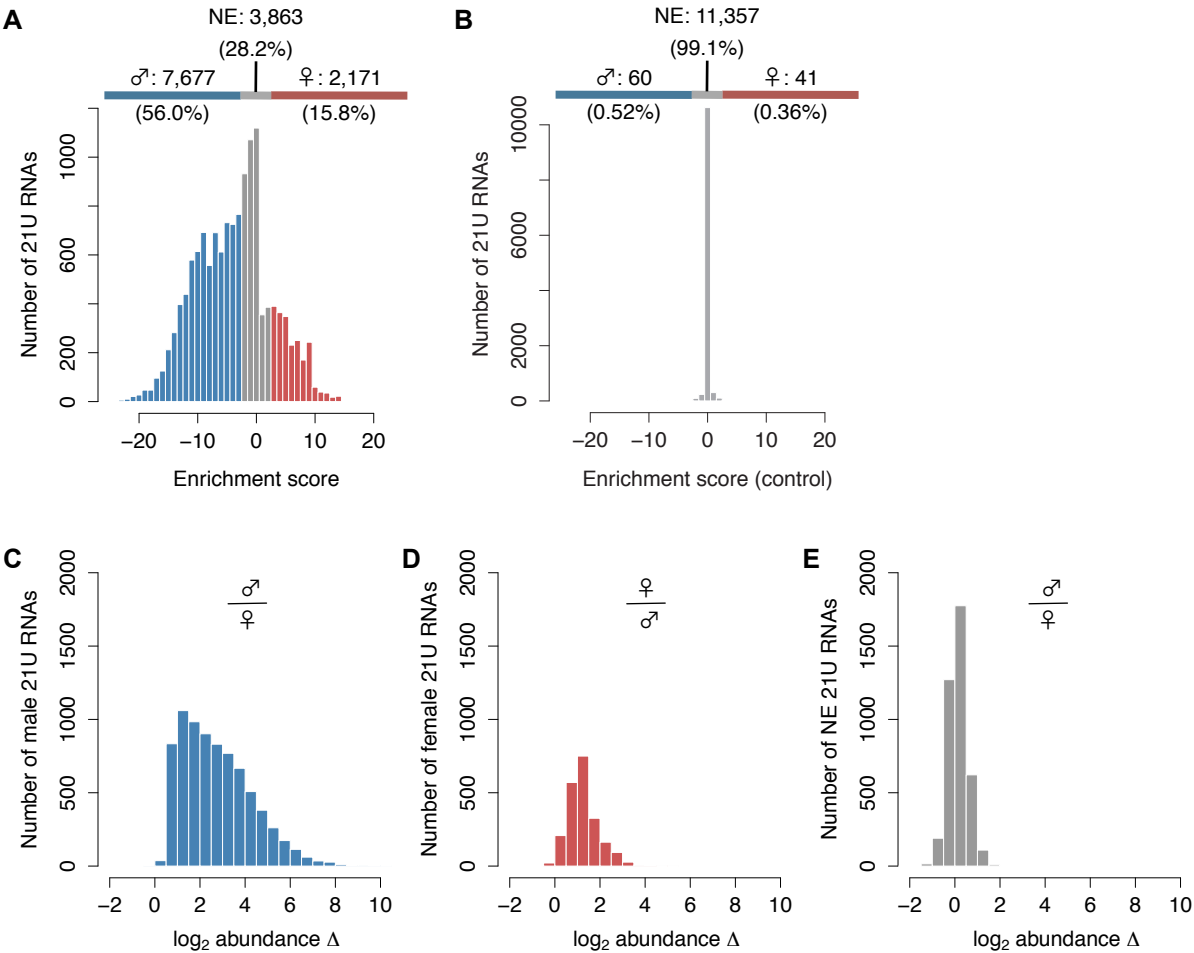

Supplement: Figure S1 — Computational identification of male and female germline-enriched 21U RNAs. (A) Enrichment Score calculations performed on 17 small RNA sequencing libraries classify a majority of 21U RNAs as male (blue) or female (red) germline-enriched. Non-enriched (NE) 21U RNAs, grey. Numbers indicate percent of 13,711 21U RNAs analyzed. (B) Enrichment Score calculations performed on control data classify <1% of 21U RNAs as male or female germline-enriched indicating a 1% false discovery rate. Numbers indicate percent of 11,458 21U RNAs analyzed. (C) Male 21U RNAs are more abundant in male libraries. Average relative abundance of each male 21U RNA was calculated between each of the 23 male∶female library comparisons. (D) Female 21U RNAs are more abundant in female libraries. Average relative abundance of each male 21U RNA was calculated between each of the 23 female∶male library comparisons. (E) Non-enriched 21U RNAs are equally abundant in male and female libraries. (PDF) [file pgen.1003392.s002.pdf]

Figure S2

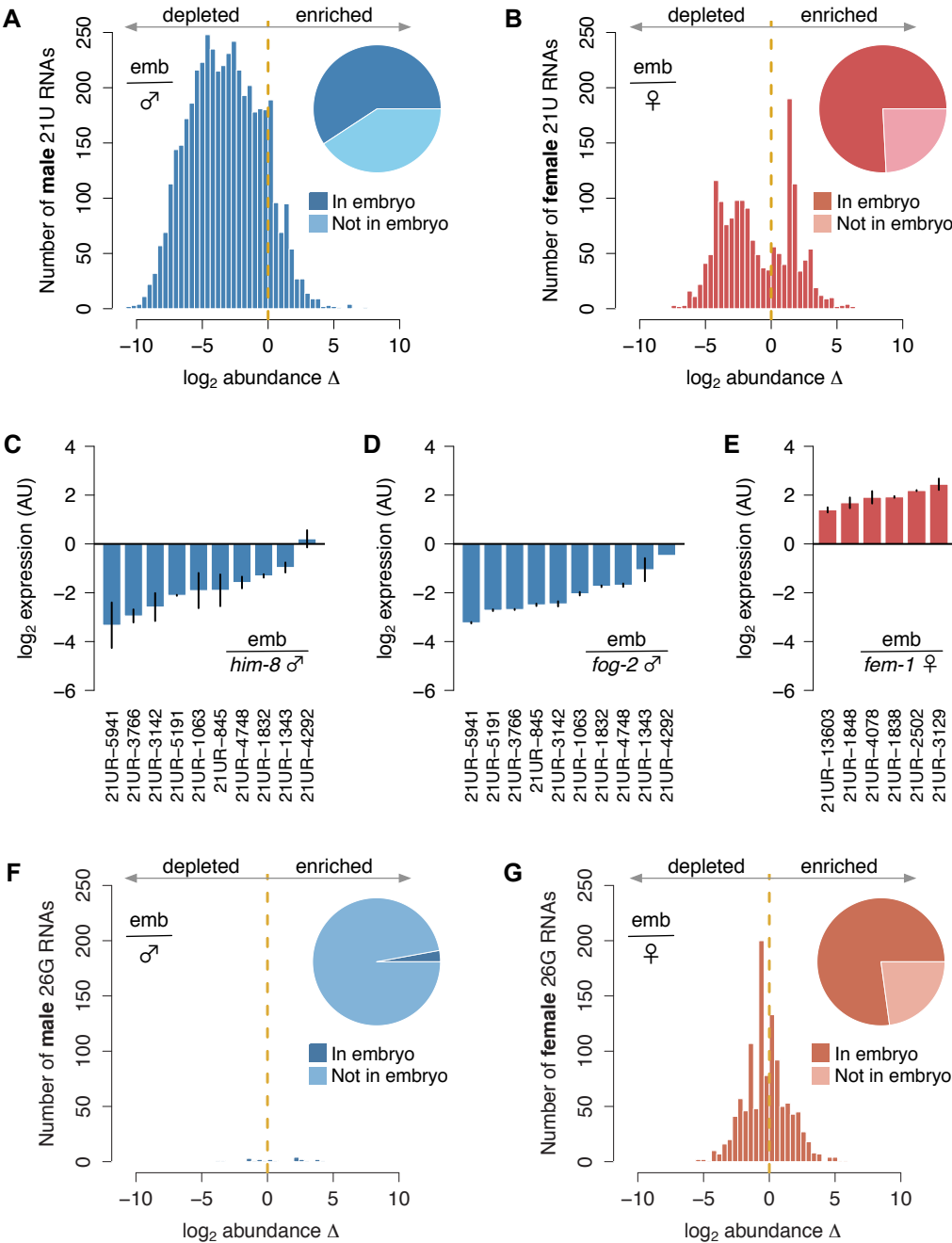

Supplement: Figure S2 — Female 21U RNAs are preferentially abundant in embryo. (A) Relative male 21U RNA abundance is decreased in embryo. Average relative abundance of each male 21U RNA was calculated between each of 5 male and 4 mixed stage embryo libraries. Dotted line indicates equal male and embryo abundance. Pie chart depicts proportion of male 21U RNAs with reads in at least one embryo library (dark blue). (B) A population of female 21U RNAs shows increased abundance in embryo. Average relative abundance of each female 21U RNA was calculated between each of 1 female and 4 mixed stage embryo libraries. Pie chart depicts proportion of female 21U RNAs with reads in at least one embryo library (dark red). (C,D) Taqman RT-qPCR analysis corroborates male 21U RNA depletion in embryo. Expression of representative male 21U RNAs was assayed by Taqman in him-8(e1489) (C) and fog-2(q71) (D) male animals and N2 embryos. Error bars represent ±1 SD from two biological replicates. (E) Taqman RT-qPCR analysis corroborates female 21U RNA enrichment in embryo. Expression of representative female 21U RNAs was assayed by Taqman in fem-1(hc17) female animals and N2 embryos. (F) Male germline-enriched 26G RNAs are generally absent in embryo. Average relative abundance of each male 26G RNA was calculated between each of 4 male and 4 mixed stage embryo libraries. (G) Female germline-enriched 26G RNAs are robustly expressed in embryo. Average relative abundance of each female 26G RNA was calculated between each of 1 female and 4 mixed stage embryo libraries. (PDF) [file pgen.1003392.s003.pdf]

Figure S3

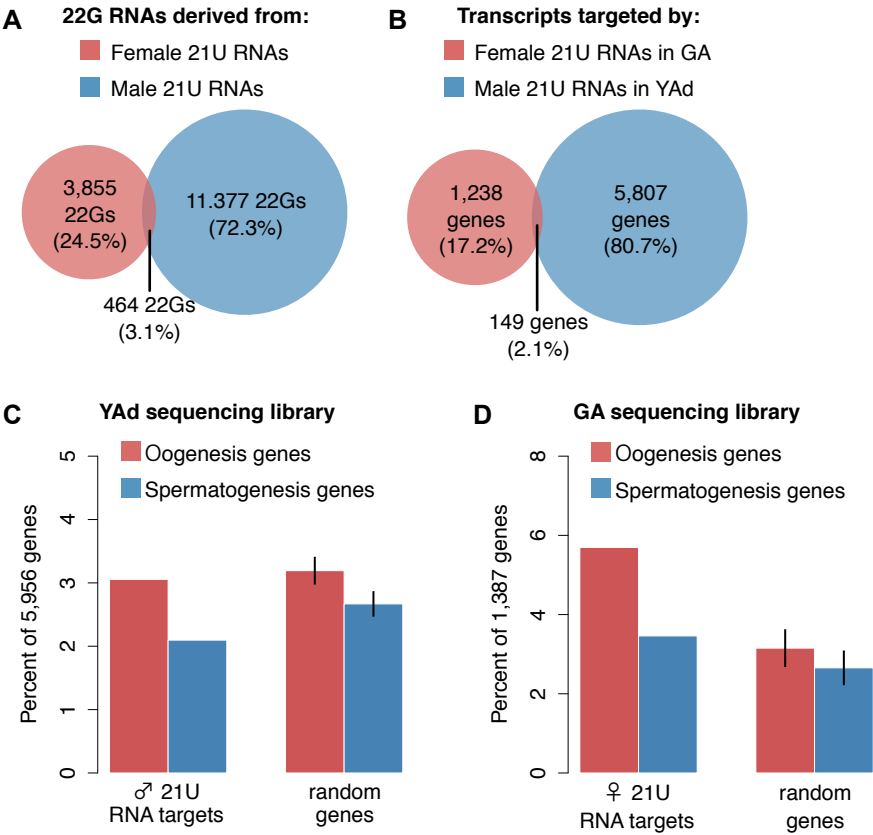

Supplement: Figure S3 — 21U RNAs target significantly non-overlapping sets of genes. (A) 22G RNAs are almost exclusively derived from either male or female 21U RNAs, but not both. The number of unique 22G RNAs derived from both male and female 21U RNAs is significantly less than expected if 22G RNAs are selected at random (Fisher's exact test, p = 1.2e−02). (B) Male and female 21U RNAs target significantly fewer overlapping genes compared to selecting random sets of genes (Fisher's exact test, p = 7.7e−13). (C) 5,956 genes targeted by male 21U RNAs in young adult (YAd) animals are depleted of spermatogenesis genes compared to a random set of 5,956 genes. (D) 1,387 genes targeted by female 21U RNAs in gravid adult (GA) animals are enriched for oogenesis genes compared to a random set of 1,387 genes. (PDF) [file pgen.1003392.s004.pdf]

Figure S4

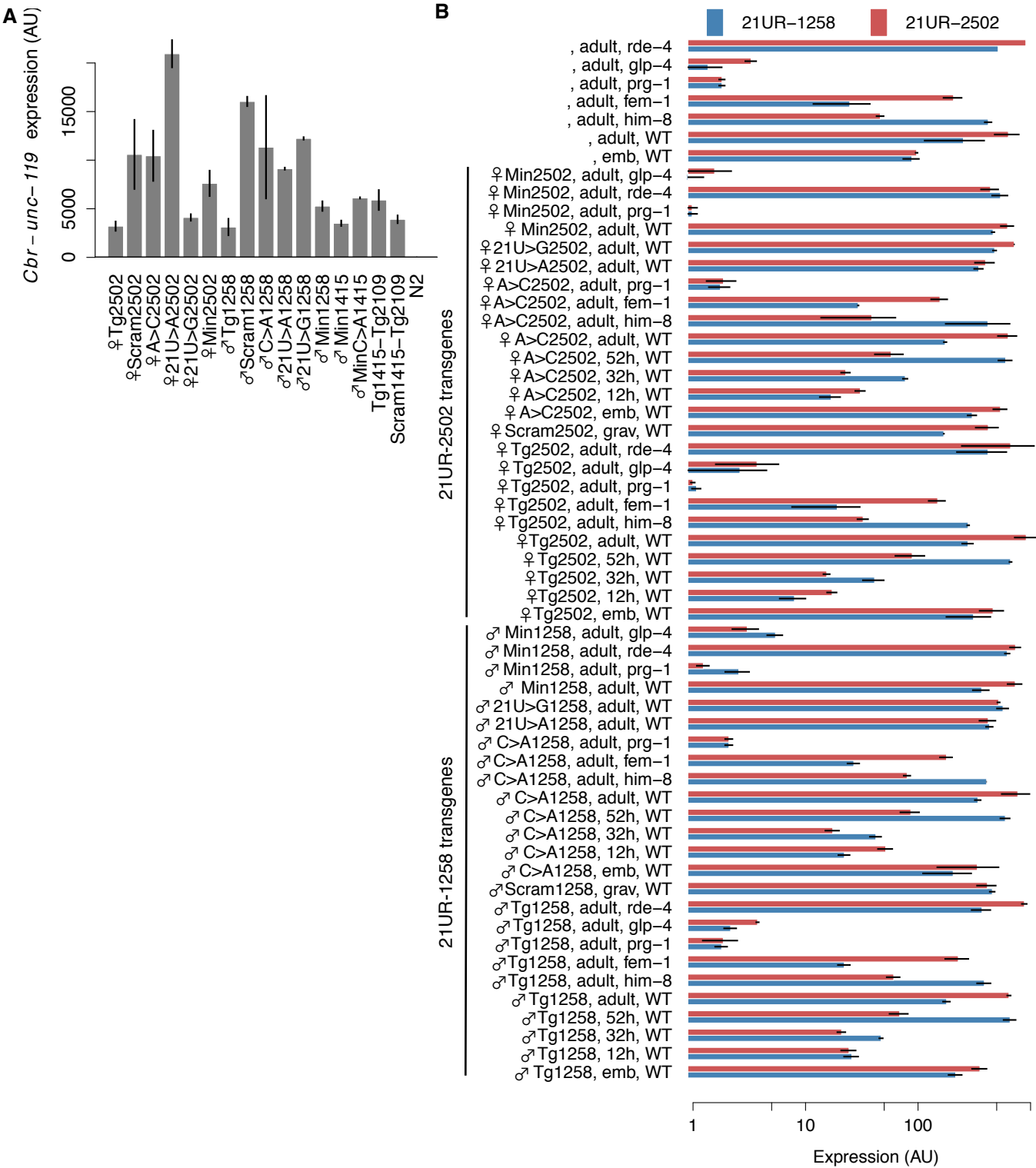

Supplement: Figure S4 — Transgenic array expression varies across transgenes. (A) Levels of Cbr-unc-119 mRNA in adult animals were assayed by RT-qPCR for all transgenes and normalized to act-1 mRNA levels. (B) Expression of transgenic 21UR-synth does not affect expression of endogenous 21U RNA counterparts. Endogenous ♂21UR-1258 and ♀21UR-2502 levels were assayed by Taqman RT-qPCR and normalized to microRNA miR-1 levels in all samples. (PDF) [file pgen.1003392.s005.pdf]

Figure S5

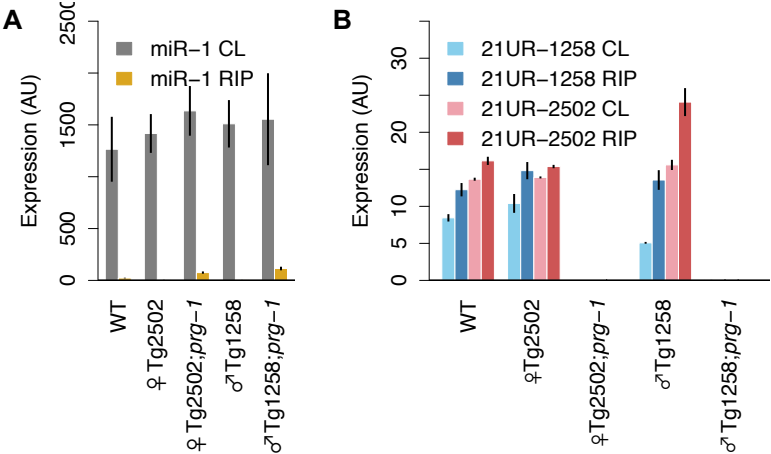

Supplement: Figure S5 — 21U RNAs are specifically immunoprecipitated with PRG-1 complexes. (A) anti-PRG-1 antibody does not immunprecipitate microRNA miR-1. (B) 21UR-synth expression does not interfere with association of endogenous 21U RNAs with PRG-1. (PDF) [file pgen.1003392.s006.pdf]

Figure S6

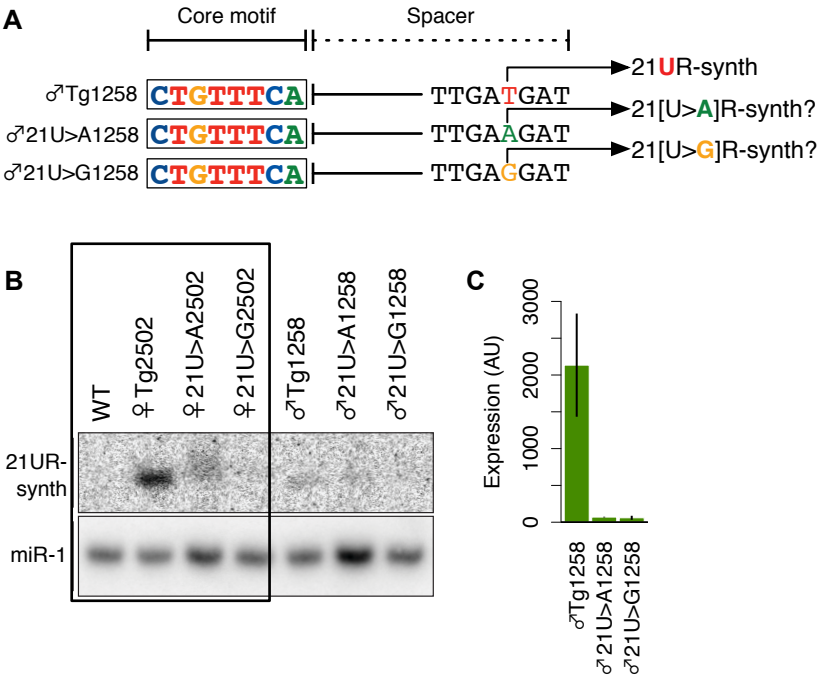

Supplement: Figure S6 — 21U RNA expression requires a 5′ genomic thymidine. (A) Schematic of transgenes encoding 21UR-synth with different 5′ nt. (B–C) Mutation of the 5′ genomic thymidine disrupts expression of 21UR-synth by northern blot (B) and Taqman assay (C). WT and ♀Tg2502 lanes in (B) are repeated from Figure 5B for clarity. (PDF) [file pgen.1003392.s007.pdf]

**Figure S7**

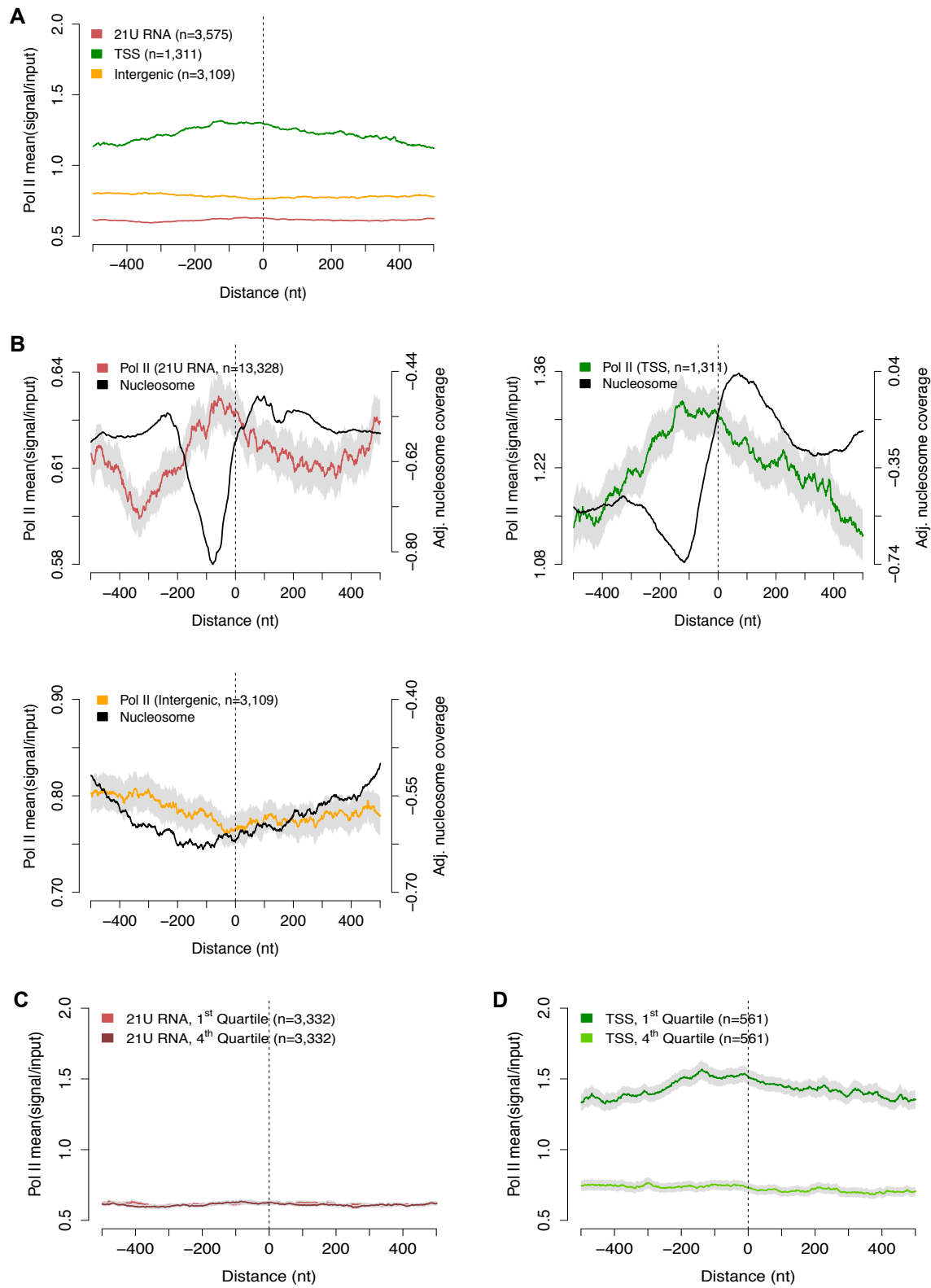

Supplement: Figure S7 — RNA polymerase II occupancy at 21U RNA loci is below background level. (A) Average Pol II occupancy in a young adult library of 21U RNA loci expressing 21U RNAs with at least 5 RPM (red), transcriptional start sites (TSS) expressing transcripts with at least 5 FPKM (green), and randomized intergenic regions (yellow). Only regions on ChrIV were assayed (B) Pol II occupancy as described in (A) but independently scaled for each transcript type and plotted with average nucleosome occupancy (black line). Grey error bands: SEM. (C) Average Pol II occupancy of 21U RNA loci as (B) but showing the top 25% 21U RNAs by abundance (1st quartile) and the bottom 25% (4th quartile) separately. 21U RNAs on all chromosomes are shown. (D) Same as (E) but showing top and bottom 25% of TSS by transcript abundance. (PDF) [file pgen.1003392.s008.pdf]

**Figure S8**

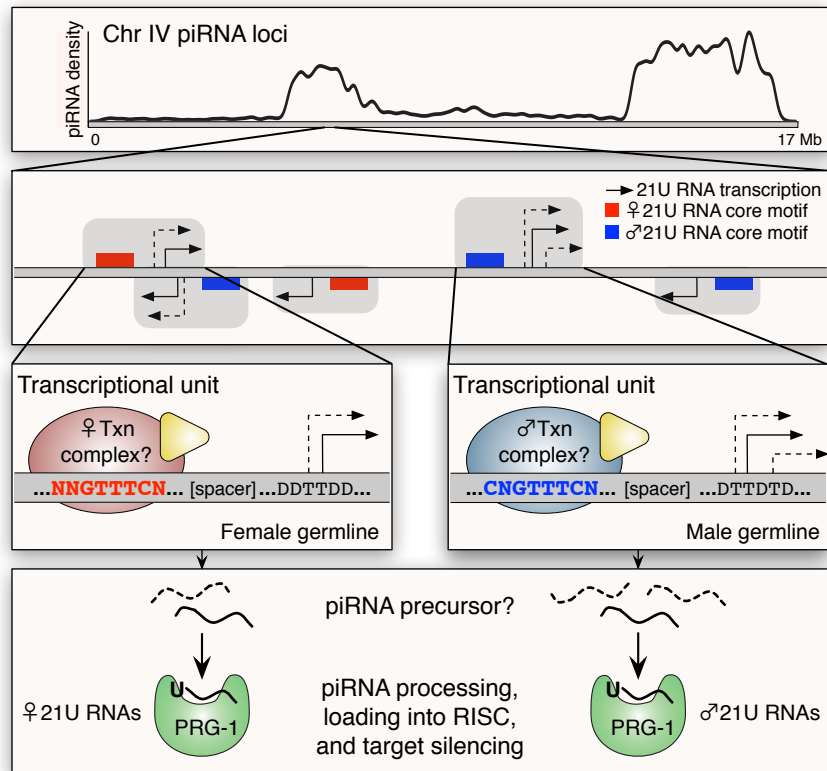

Supplement: Figure S8 — Model of 21U RNA expression. (PDF) [file pgen.1003392.s009.pdf]
