# Supplementary material for: A Conserved Upstream Motif Orchestrates Autonomous, Germline-Enriched Expression of Caenorhabditis elegans piRNAs
Source: PLoS Genet. 2013 Mar 14;9(3):e1003392. doi: 10.1371/journal.pgen.1003392 (PMC3597512; doi:10.1371/journal.pgen.1003392)
Supplement: Table S1 — Descriptions of small RNA sequencing libraries used in this study. GEO Accessions for datasets and libraries used are listed. Libraries generated using 5′-monophosphate-dependent (Dep) or -independent (Indep) RNA extraction protocols are indicated along with how the library was used in this study (“Use” column). (PDF) [file pgen.1003392.s010.pdf]

Table S1. Descriptions of small RNA sequencing libraries used in this study.

GEO Accessions											
Dataset	*Library*	*Genotype*	*Developmental Stage*	*Extraction protocol*	*Sequencing platform*	*Raw reads*	*Mapped reads*	*%*	*21U RNA reads*	*%*	*Use*
GSE20341	GSM510085	N2	mixed-stage embryos	Dep	Illumina	4,661,109	3,721,872	79.8	31,837	0.9	Embryo analysis
	GSM509932/ GSM510089	*him-8(e1489)*	isolated sperm-atogenic cells	Dep	Illumina/454	9,596,732	1,312,607	13.7	25,024	1.9	Germline enrichment
	GSM509933/ GSM510090	*fer-1(hc1)*	purified unfertilized oocytes	Dep	Illumina/454	6,488,731	2,165,341	33.4	47,731	2.2	Germline enrichment
GSE11738	GSM297742	N2	mixed-stage embryos	Dep	Illumina	2,730,450	2,382,829	87.3	52,072	2.2	Embryo analysis
	GSM297751	N2	young adult	Dep	Illumina	3,533,717	3,169,078	89.7	333,587	10.5	Random control, spacer analyses
	GSM297755	*prg-1(tm872)*	young adult	Dep	Illumina	3,588,293	3,303,711	92.1	2,577	0.1	21U RNA filtering
	GSM297753	*fog-2(q71)*	young adult	Dep	Illumina	3,387,268	2,960,986	87.4	297,715	10.1	Germline enrichment
GSE18215	GSM455395	*fem-1(hc17)*	purified oocytes	Indep	Illumina	8,496,639	7,575,752	89.2	53,848	0.7	Germline enrichment
GSE19414	GSM503834	*fem-1(hc17)*	adult	Indep	Illumina	389,636	369,130	94.7	224	0.1	Germline enrichment
	GSM503842	*mut-16(mg461); fem-3(q20)*	isolated sperm-atogenic cells	Dep	Illumina	425,438	399,905	94.0	94,863	23.7	Germline enrichment
	GSM503843	*rrf-3(pk1426); fem-3(q20)*	isolated sperm-atogenic cells	Dep	Illumina	650,621	608,159	93.5	46,864	7.7	Germline enrichment
GSE17153	GSM427297	N2	mixed-stage embryos	Dep	Illumina	2,159,213	1,681,110	77.9	6,926	0.4	Embryo analysis
GSE13339	GSM336052	N2	mixed-stage embryos	Dep	Illumina	6,391,734	2,746,387	43.0	29,378	1.1	Embryo analysis
	GSM336086	*dpy-28(y1); him-8(e1489)*	young adult	Dep	Illumina	3,653,638	1,357,061	37.1	21,778	1.6	Germline enrichment
GSE18729	GSM465244	*alg-3(tm1155); alg-4(ok1041); fog-2(q71)*	adult	Indep	Illumina	3,216,031	3,003,318	93.4	237,635	7.9	Germline enrichment
	GSM465245	*fog-2(q71)*	adult	Indep	Illumina	821,513	757,771	92.2	45,592	6.0	Germline enrichment
	GSM465246	*fog-2(q71)*	adult	Indep	Illumina	2,740,511	2,562,914	93.5	157,991	6.2	Germline enrichment
	GSM465247	*fem-3(q20)*	isolated spermatids	Indep	Illumina	10,478,418	7,131,378	68.1	256,516	3.6	Germline enrichment
GSE18429	GSM459328	*fem-3(q20)*	isolated sperm-atogenic cells	Indep	Illumina	375,816	341,766	90.9	8,029	2.3	Germline enrichment
	GSM459329	*rrf-3(pk1426); him-8(e1489)*	young adult	Indep	Illumina	1,756,561	1,673,756	95.3	15,702	0.9	Germline enrichment
	GSM459330	*him-8(e1489)*	young adult	Indep	Illumina	1,709,934	1,614,576	94.4	3,515	0.2	Germline enrichment
	GSM459331	*rrf-3(pk1426); him-8(e1489)*	young adult	Indep	Illumina	1,492,360	1,366,804	91.6	9,238	0.7	Germline enrichment
	GSM459332	*him-8(e1489)*	young adult	Indep	Illumina	755,623	695,239	92.0	2,191	0.3	Germline enrichment
