# Supplementary material for: A Conserved Upstream Motif Orchestrates Autonomous, Germline-Enriched Expression of Caenorhabditis elegans piRNAs
Source: PLoS Genet. 2013 Mar 14;9(3):e1003392. doi: 10.1371/journal.pgen.1003392 (PMC3597512; doi:10.1371/journal.pgen.1003392)
Supplement: Table S2 — Welch's t-test p-values for all abundance comparisons between 21U RNAs with different core motifs. Highlighted are p-values <0.01. Identity of the 5′ nt corresponding to higher 21U RNA abundance is indicated below each significant p-value. All t-tests are two-tailed. Comparisons of abundances in 5′-monophosphate-dependent and -independent libraries were performed separately. (PDF) [file pgen.1003392.s011.pdf]

Table S2. Welch's *t*-test p-values for all abundance comparisons between 21U RNAs with different core motifs.

Library type	*Enrichment classification*	*A vs. C*	*A vs. G*	*A vs. T*	*A vs. N*	*C vs. G*	*C vs. T*	*C vs. N*	*G vs. T*	*G vs. N*	*T vs. N*
Male (5'-mP_i-Dep.)	Male	**2.4E-13**<br>**C**	3.5E-01<br>-	**3.5E-03**<br>**A**	**2.0E-08**<br>**A**	**1.2E-03**<br>**C**	**6.0E-11**<br>**C**	**1.3E-56**<br>**C**	3.6E-01<br>-	2.2E-01<br>-	7.7E-01<br>-
	Non-enriched	1.9E-01<br>-	4.2E-01<br>-	2.8E-01<br>-	**3.3E-05**<br>**A**	1.5E-01<br>-	3.0E-02<br>-	**2.1E-12**<br>**C**	9.0E-01<br>-	3.1E-01<br>-	5.3E-02<br>-
Female (5'-mP_i-Dep.)	Female	**6.1E-03**<br>**A**	2.4E-02<br>-	9.8E-01<br>-	1.4E-01<br>-	4.0E-01<br>-	1.9E-02<br>-	6.8E-02<br>-	3.1E-02<br>-	9.6E-02<br>-	2.2E-01<br>-
	Non-enriched	**6.0E-03**<br>**A**	5.0E-01<br>-	9.3E-01<br>-	**4.6E-03**<br>**A**	5.9E-01<br>-	3.6E-02<br>-	9.5E-01<br>-	4.9E-01<br>-	5.7E-01<br>-	3.2E-02<br>-
Male (5'-mP_i-indep.)	Male	**4.06E-09**<br>**C**	2.48E-01<br>-	**6.40E-04**<br>**A**	**5.14E-07**<br>**A**	**1.09E-03**<br>**C**	**1.90E-11**<br>**C**	**8.21E-45**<br>**C**	2.72E-01<br>-	3.28E-01<br>-	6.79E-01<br>-
	Non-enriched	**4.04E-03**<br>**A**	8.76E-01<br>-	9.41E-01<br>-	**1.84E-04**<br>**A**	2.59E-01<br>-	2.99E-02<br>-	2.73E-01<br>-	8.49E-01<br>-	1.33E-01<br>-	**5.31E-03**<br>**T**
Female (5'-mP_i-indep.)	Female	**1.53E-05**<br>**A**	**2.62E-03**<br>**A**	4.17E-01<br>-	**4.44E-04**<br>**A**	4.20E-01<br>-	**5.98E-03**<br>**T**	1.06E-01<br>-	1.72E-02<br>-	1.18E-01<br>-	6.69E-02<br>-
	Non-enriched	**4.12E-06**<br>**A**	8.19E-01<br>-	8.04E-01<br>-	**6.90E-04**<br>**A**	9.71E-02<br>-	**1.99E-03**<br>**T**	1.10E-01<br>-	9.42E-01<br>-	2.40E-01<br>-	2.63E-02<br>-

P-values colored gold are significant at $p<0.01$. Letters below p-values indicate which nt corresponds to higher abundance and are colored to match Weblogos.
Boxed p-values/letters show that male 21U RNAs with 5'-cytidine motifs are more abundance than any other 5'-nt.
mPi-indep: monophosphate independent; mPi-dep: monophosphate dependent.
